# Biodegradable Zein-Based Biocomposite Films for Underwater Delivery of Curcumin Reduce Thermal Stress Effects in Corals

**DOI:** 10.1021/acsami.3c01166

**Published:** 2023-06-28

**Authors:** Marco Contardi, Marta Fadda, Valerio Isa, Yohan D. Louis, Andrea Madaschi, Sara Vencato, Enrico Montalbetti, Laura Bertolacci, Luca Ceseracciu, Davide Seveso, Silvia Lavorano, Paolo Galli, Athanassia Athanassiou, Simone Montano

**Affiliations:** † Department of Earth and Environmental Sciences (DISAT), University of Milan − Bicocca, Milan 20126, Italy; ‡ MaRHE Center (Marine Research and High Education Center), Magoodhoo Island, Faafu Atoll 12030, Republic of Maldives; § Smart Materials, Istituto Italiano di Tecnologia, Genova 16163, Italy; ∥ Materials Characterization Facility, Istituto Italiano di Tecnologia, Genova 16163, Italy; ⊥ Costa Edutainment SpA - Acquario di Genova, Genova 16128, Italy; # Dubai Business School, University of Dubai, Dubai 14143, United Arab Emirates

**Keywords:** coral reefs, coral bleaching, antioxidants, underwater delivery, biocomposites

## Abstract

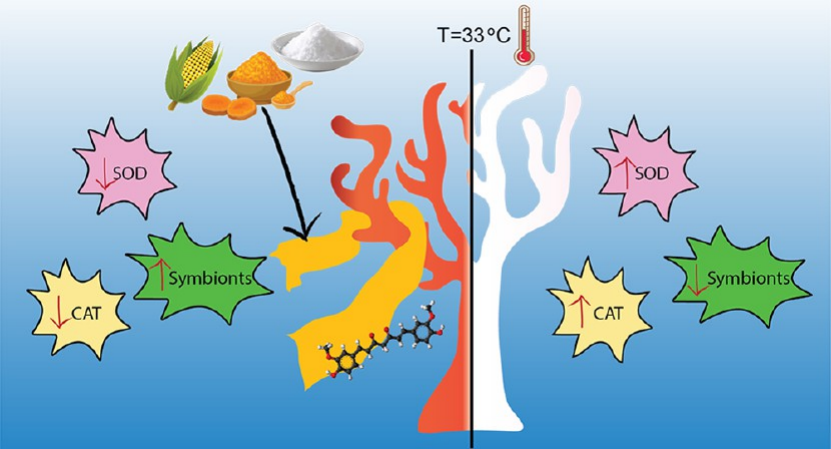

Massive coral bleaching episodes induced by thermal stress are one of the first causes of coral death worldwide. Overproduction of reactive oxygen species (ROS) has been identified as one of the potential causes of symbiosis breakdown between polyps and algae in corals during extreme heat wave events. Here, we propose a new strategy for mitigating heat effects by delivering underwater an antioxidant to the corals. We fabricated zein/polyvinylpyrrolidone (PVP)-based biocomposite films laden with the strong and natural antioxidant curcumin as an advanced coral bleaching remediation tool. Biocomposites’ mechanical, water contact angle (WCA), swelling, and release properties can be tuned thanks to different supramolecular rearrangements that occur by varying the zein/PVP weight ratio. Following immersion in seawater, the biocomposites became soft hydrogels that did not affect the coral’s health in the short (24 h) and long periods (15 days). Laboratory bleaching experiments at 29 and 33 °C showed that coral colonies of *Stylophora pistillata* coated with the biocomposites had ameliorated conditions in terms of morphological aspects, chlorophyll content, and enzymatic activity compared to untreated colonies and did not bleach. Finally, biochemical oxygen demand (BOD) confirmed the full biodegradability of the biocomposites, showing a low potential environmental impact in the case of open-field application. These insights may pave the way for new frontiers in mitigating extreme coral bleaching events by combining natural antioxidants and biocomposites.

## 1 Introduction

Coral reefs are unique ecosystems characterized by vast biodiversity, being the habitat for millions of marine species.^1^ On top of that, coral reefs support the livelihood of millions of people, and they are the primary resource of numerous local economies worldwide, through fishing, tourism, and coastal protection. Nowadays, though, coral reefs are undergoing widespread destruction due to human-induced climate and environmental changes such as increased ocean temperatures. In addition, pollution from plastics, overfishing, ocean acidification, and diseases are negatively affecting the survival of coral reefs.^2−4^

Coral bleaching, the process whereby large extents of corals rapidly pale through the loss of their algal endosymbionts, represents one of the most worrying reasons for coral reef loss.^5,6^ The symbiosis interruption leads to coral cover losses, causing coral morbidity and mortality, dramatic changes to coral community composition, and rapid reorganization of coral reef-fish communities. Severe coral bleaching events, including the 2014–2017 global coral-bleaching event, the third in the last 20 years, occur fivefold faster than in the past, killing reef organisms over thousands of square kilometers.^7^ These massive events are provoked by changes to environmental factors such as enhanced UV-light exposure, increased salinity, presence of iron and other heavy metals, unbalance of inorganic nutrients such as CO_2_, and variations of the nitrogen-to-phosphate ratio.^8−11^ Still, the increase in sea surface temperatures (SSTs) due to global warming is considered the principal cause of the large-scale loss of reefs.^9^ In addition, bleaching susceptibility depends on the intrinsic tolerance of coral species, the duration and intensity of the stress, and the coral microbiome’s status,^11−14^ making extremely challenging the prediction of bleaching events and the evaluation of bleaching effects and damages.

During extreme heat wave events, several alterations occur inside the corals and the molecular reasons, which lead to coral bleaching, are still under investigation.^15^ However, reactive oxygen/nitrogen species (ROS/RNS) generated during exposure to environmental stressors have been described as the primary toxic agents at the cellular level, responsible for the disruption of symbiosis between the polyp and the microalgae zooxanthellae (Symbiodiniaceae). In response to heat stress and increased ROS and RNS concentrations, the polyp and algae trigger various molecular pathways that work together to counteract oxidative stress. Specifically, these pathways lead to the synthesis and expression of enzymes, proteins, and stress factors, as for example, the enzymes superoxide dismutase (SOD), glutathione reductase, and catalase (CAT), heat-shock proteins (HSPs), and the nuclear factor kappa B (NF-kB).^16,17^

Recently, researchers have developed various methodologies to mitigate coral bleaching events. For instance, artificial upwelling of colder deep water to the surface has been designed to balance the increasing temperature and avoid diffused coral bleaching.^18,19^ A geoengineering approach called “shading” has also been evaluated to reduce the amount of solar light that reaches the reef. By spraying microscopic salt particles into low-lying marine clouds, it is possible to increase the scattering and reflectivity of the sunlight from the clouds and, thus, reduce the amount of solar radiation energy that warms marine waters.^20,21^ Another pioneering treatment based on the isolation and culture of bacteria from the coral microbiome showed promising results.^22^ Probiotics were dispersed in an aqueous solution and then administered to corals in tanks during a simulated seawater heating event. Corals treated with probiotics tolerated and handled the thermal stress and recovery phases better. Although probiotics are a promising remedy, their isolation, sequencing, and culture are expensive and time-consuming, thus limiting their scalable production. Moreover, the lack of a vehicle that ensures proper delivery on corals could drastically reduce the efficacy of probiotics due to their dispersion and dilution in the open sea, calling for further efforts to release this technology on a large scale.

In the last years, biomaterials and biocomposites have emerged as new tools for building new sustainable, eco-friendly, and biocompatible strategies for coral restoration and healing. In this regard, 3D objects are considered safety solutions for supporting coral reef restoration.^23^ Specifically, 3D-printed artificial coral skeletons based on poly(lactic acid) ensured fast growth of coral fragments attached to their surface, thus accelerating the transplantation to the reef.^24^ 3D-printed soft hydrogels that mimic the corallite architecture, including optical and mechanical properties, were also fabricated. These “bionic corals” allowed proper growth of green microalgae inside their matrices, opening a way to produce incubators and bioreactors for coral algae cultivation.^25,26^ In addition, biocomposites in the form of films can act as scaffolds for coral larvae.^27^ Contardi et al. reported the first example of biomaterials for delivering drugs in coral wounds.^28^ The therapy was based on an application in two steps, combining an adhesive bilayer film loaded with antibiotics and antiseptics and a thermoplastic ε-caprolactone-*p*-coumaric acid co-polymer to seal the area. The treatment allowed confining the drugs in the wound area, avoiding their dispersion into the sea. New coral tissue also grew on the biomaterials, highlighting the high degree of biocompatibility of these polymers toward corals.

ROS and RNS production and diffusion are commonly inhibited by synthetic and natural antioxidant molecules due to their capacity to scavenge and stabilize free radicals in their chemical structure.^29^ At the same time, natural antioxidants can modulate different molecular pathways inside cells in response to oxidative stress,^30,31^ making them ideal candidates as potential drugs for contrasting coral bleaching. Among them, curcumin is one of the most studied and used. It is a natural molecule in the *Curcuma longa* plant and is employed in several fields, such as therapeutic agents in biomedicine, food packaging, pH indicators, and functional coatings.^32−35^ Moreover, curcumin has been proven to regulate SOD, HSPs, and NF-kB synthesis and action in human cells.^36−38^ These targets, as previously described, are the same mediators for the bleaching response within the coral tissue.

When administering antioxidants to human beings, they are usually loaded in formulations, which improve their stability within the body, reach the correct site of action, and have an adequate release profile.^39−41^ In a similar concept, this work aimed to produce environmentally low-impact, biodegradable, biocompatible biocomposite films based on zein and poly(vinylpyrrolidone) to deliver the antioxidant molecule curcumin into corals. Zein is a protein present in maize with a high content of apolar amino acids such as leucine, alanine, and proline. It has been used in several fields, from pharmaceutics, as a natural and biocompatible polymer, to food packaging and coatings as an eco-friendly alternative to current plastic-based materials.^42^ PVP is a hydrophilic polymer widely used in pharmaceutics and cosmetics for its biocompatibility and capacity to quickly disaggregate in water and improve drug solubility by altering their crystallinity.^43^

The physicochemical properties of the fabricated systems and their underwater resistance and biocompatibility when applied to corals have been extensively described. The efficacy of curcumin in preventing coral bleaching and related cellular damages has been investigated by exposing the coral *Stylophora pistillata* to three fixed temperatures, 25, 29, and 33 °C, to simulate heat-stress events. The concentration of chlorophylls (Chls), which are biomarkers for the symbiosis status, and the activity of the antioxidant enzymes SOD and CAT in the stressed corals have been quantified. The results demonstrated how advanced biocomposites loaded with antioxidants can be a new weapon for effectively preventing coral bleaching.

## 2 Experimental Section

### 2.1 Chemicals and Materials

Zein (average MW = 20 kDa), polyvinylpyrrolidone (PVP) (MW = 360 kDa), glycerol, curcumin from *Curcuma longa* (turmeric) powder, methanol, dimethyl sulfoxide (DMSO) (≥99.5%), and phosphate-buffered saline (PBS) were purchased from Sigma-Aldrich and used without further purification.

### 2.2 Preparation of Films

Zein powder was dissolved in 6 mL of DMSO and stirred for 6 h at room temperature. Meanwhile, a methanol solution with glycerol (10% w/w with respect to the total amount of polymers) was prepared and added to the zein solution. PVP and curcumin powders were added to the mix and left under a stirrer for 24 h. Different weight ratios between zein and PVP were investigated, while curcumin was used at the fixed concentration of 2.4% with respect to the total amount of polymers. Afterward, the solutions were poured into square Teflon dishes (9 × 9 cm) and kept in the dark under an aspirated hood at ambient conditions (16–20 °C and 40–50% RH) for 3 h to eliminate the major part of the solvents (methanol and DMSO) and avoid any formation of bubbles. Finally, the square Teflon dishes were put on a hot plate at 95 °C for 48 h to eliminate the residual solvents (methanol and DMSO). The compositions and labels of each prepared film are reported in Table S1.

### 2.3 Scanning Electron Microscopy (SEM)

The morphology of the obtained films was analyzed by SEM, using a variable-pressure JEOL JSM-649LA (JEOL, Tokyo, Japan) microscope equipped with a tungsten thermionic electron source and working in high vacuum mode, with an acceleration voltage of 5 kV. The specimens were coated with a 10 nm-thick film of gold utilizing the Cressington 208HR Sputter Coater (Cressington, Watford, UK).

The morphology of the Z/P 6/4 sample after swelling was obtained by SEM using a different preparation. First, the swollen materials were placed at −80 °C in a standard fridge for 24 h and then dehydrated by a 5Pascal Base Unit LIO5P4K with the fixed parameters −50 °C and 0.5–0.3 mbar. Afterward, the samples were kept in a dry environment before sputtering with gold, as described previously.

### 2.4 Attenuated Total Reflection-Fourier Transform Infrared (ATR-FTIR) Spectroscopy

Infrared spectra of the biocomposite materials were acquired by using an ATR accessory (MIRacle ATR, PIKE Technologies) with a diamond crystal coupled to an FTIR spectrometer (Vertex 70v FTIR, Bruker). All spectra were recorded between 4000 and 600 cm^–1^, with a resolution of 4 cm^–1^, accumulating 128 scans.

### 2.5 X-ray Diffraction

The physical state of the biocomposites and their pristine components were determined by X-ray diffraction spectroscopy. X-ray diffractograms were obtained using a Malvern PANalytical Empyrean X-ray diffractometer (Malvern Panalytical, Malvern, UK) equipped with a 1.8 kW Cu Kα source sealed in a ceramic tube, and a 0D Xe proportional detector with a PixCel 3D 2x2 area detector. The samples were placed on quartz support, and experiments were performed using a Cu Kα anode (λ = 1.5406 °A) operated at 45 kV and 40 mA from 5 to 65° 2θ.

### 2.6 Thermal Characterization

The thermal degradation behavior of the biocomposites and their pristine components was determined by thermogravimetric analysis (TGA) and using a TGA Q500 (TA Instruments, USA) instrument. Measurements were carried out using 3–5 mg of sample in a platinum pan under inert N_2_ flow (50 mL min^–1^) in a temperature range from 30 to 800 °C and with a heating rate of 10 °C min^–1^. The weight loss and its first derivative were acquired simultaneously as a function of time/temperature.

Differential scanning calorimetry (DSC) thermograms were acquired with the Discovery DSC 250 (TA Instruments, Waters^TM^ Division, USA) from RT to *T*_f_ under nitrogen flow (50 mL min^–1^) at 10 °C min^–1^ using non-hermetic aluminum pans. About 4 mg of the sample was used. Specimens were first heated to *T*_f_ to release moisture and cooled to RT, and finally, the temperature was ramped to 130 °C for the Z/P 10/0, 6/4, and 0/10 samples; 150 °C for the PVP + Gly sample; 160 °C for the zein and Zein + Gly samples; and 200 °C for the PVP sample. The maximum temperature of the ramp was decided considering the results of the TGA (methods and results are reported in the SI and Figures S7A and S7B). The glass transition temperature (*T*_g_) was calculated using the inflection method.

### 2.7 Mechanical Properties

The mechanical properties of zein/PVP-based samples were determined by uniaxial tension tests on a dual-column universal testing machine Instron 3365 (Instron, Norwood, Massachusetts, USA). Biocomposites were cut in dog bone specimens (at least 10 for each sample) with a width of 4 mm and an adequate length of 25 mm. Displacement was applied at a rate of 10 mm min^–1^. Young’s modulus, tensile stress at maximum load, and elongation at break were calculated from the stress–strain curves. Tests were conducted at 25 °C and in two different conditions of humidity, namely, at 0 and 84% R.H. For the 0% R.H., the samples were stored in a closed chamber for 48 h with dried silica beads, the chamber of 84% R.H. with an oversaturated solution of KCl.^44^

The mechanical properties of biocomposite materials were characterized by nanoindentation with the Chiaro Nanoindenter (Optics11, Netherlands), working in displacement control. The system uses cantilever probes to apply controlled indentation to the samples, while evaluating the reaction force from the cantilever deflection, measured by interferometry. Before the analysis, the samples were kept for 24 h in seawater to assess their mechanical properties after the swelling.

Two probes were used for the measurements, depending on the samples’ stiffness: ≈5 N/m stiffness, ≈25 μm radius for softer samples (Z/P 5/5, 4/6, 2/8), ≈5 N/m stiffness, and ≈10 μm radius for stiffer samples (Z/P 10/0, 8/2, 6/4). Tests were conducted in liquid in displacement control; displacement was applied and removed at 5 μm/s. Indentation curves, limited to the first 2–3 μm to avoid the influence of the substrate, were fitted with the classical Hertzian model, from where the Young’s modulus was extracted.

### 2.8 Water Contact Angle

Water contact angles (WCAs) of the zein/PVP-based films were measured by using an OCA 20 contact angle goniometer (DataPhysics, Instruments GmbH, Filderstadt, Germany) at room temperature. Deionized water droplets of 5 μL were laid on the surface, and the contact angle was calculated from the side view with the help of the software. To ensure repeatability, six different measurements were taken for each sample.

### 2.9 Swelling Properties

The films’ aqueous liquid absorption and erosion behaviors were evaluated by immersing ≈ 80 mg of the samples in 12 mL of salty water to mimic the underwater condition in terms of pH salts and composition. Before the experiment, the samples were kept in a dry chamber with silica beads at 0% R.H. to remove all the absorbed humidity. Samples were weighed after 6 and 24 h, and the excess water was removed by placing the samples on filter paper. The swelling degree (SD, %) was calculated by using eq 1:
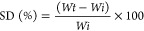
1where *Wt* corresponds to the weight of the swollen sample at the time *t* and *Wi* is the initial dry weight. Measurements were performed in triplicate.

### 2.10 Drug Release Studies

The curcumin release from the Z/P films was measured using a Varian Cary 6000i Scan UV–visible spectrophotometer (Walnut Creek, California, USA). Curcumin in water has a characteristic UV absorbance peak at 426 nm. A curcumin calibration curve was constructed to extrapolate the molar extinction coefficient, leading to ε = 1800 cm^–1^ M^–1^. At time zero, *t* = 0 s, square samples of 1 × 1 cm and ≈5 mg were placed in falcon tubes containing 13 mL of PBS at 25 °C and then measurements were performed at specific time intervals. Before each measure, the falcon tubes were gently agitated to have a good dispersion of the released compounds in the solvent medium. At each time point, 3 mL of solution was taken out and replaced with the same amount of fresh medium. Likewise, the curcumin release profile of the Z/P 6/4 film was evaluated at 29 and 33 °C to simulate a condition of increased seawater temperature. All measurements were taken so that sinking conditions were maintained, and the validity of the Beer–Lambert law was reassured. The experiments were carried out in triplicate, and the data were expressed as a cumulative percentage. All falcon tubes were sealed with Parafilm tape to ensure no water evaporation occurred.

### 2.11 ABTS Free Radical Cation Scavenging Assay

An ABTS (2,2′-azino-bis(3-ethylbenzothiazoline-6-sulfonic acid)) free radical cation scavenging assay was performed as described in Fadda et al.^34^ ABTS radical cation (ABTS^·+^) was generated by the reaction between 7 mM ABTS water solution with 2.45 mM potassium persulfate solution in the dark at room temperature for 12–16 h. The ABTS^·+^ solution was diluted with water to obtain an absorbance of 0.80 a.u. at 734 nm. After that, circles of zein/PVP-based films cut with a circular puncher of 0.6 cm in diameter were added to 3 mL of diluted ABTS^·+^ solution. The decrease in absorbance was determined at 734 nm with a Varian Cary 6000i Scan UV–visible spectrophotometer (Walnut Creek, California, USA) at different times. All measurements were carried out in triplicate, and the results were averaged to obtain a mean value. Radical scavenging activity (RSA) was expressed as the inhibition percentage of free radicals of the sample and calculated by using eq 2:
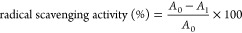
2where *A*_0_ is the absorbance value of the control radical cation solution and *A*_1_ is the absorbance value of the sample at different time points.

### 2.12 Water Vapor Permeability

Water vapor permeability (WVP) of the Z/P 6/4 film and Parafilm tape was determined at 25 °C and under 100% R.H. according to the ASTM E96 standard method. 100% R.H. was reached by placing 400 μL of deionized water in the permeation chambers of 7 mm inner diameter and 10 mm inner depth.

The samples were cut, placed on the top of the permeation chamber, and sealed through O-rings and screws. The chambers were placed in a desiccator and maintained at 0% R.H. by anhydrous silica gel.

The weight changes of the chambers were collected every hour for 8 consecutive hours to monitor the transfer of water from the chamber through the sample to the silica gel. An electronic balance (0.0001 g accuracy) recorded mass loss over time. The water mass loss of permeation chambers was plotted as a time function. The slope of each line was calculated by linear regression. Then, the water vapor transmission rate (WVTR) was determined as below (eq 3):
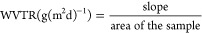
3

The WVP of the samples was calculated as follows (eq 4):
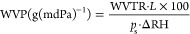
4where *L* (m) is the thickness of the sample, which was measured with a micrometer with 0.001 mm accuracy, ΔRH (%) is the percentage relative humidity gradient, and *p*_s_ (Pa) is the saturation water vapor pressure at the experimental temperature of 25 °C.^45^ Every measurement was replicated three times.

### 2.13 Biochemical Oxygen Demand

Biochemical oxygen demand (BOD) was used to investigate the biodegradability of the samples, which can be easily determined by monitoring the oxygen consumption in a closed respirometer. In detail, samples were cut into small pieces (around 5 mm side squares) and about 1 g of material was added to 432 mL of seawater as the single carbon source. Seawater was chosen to mimic actual environmental conditions. It already contained microbial consortia and the saline nutrients needed for their growth.

The experiment was conducted at room temperature inside dark glass bottles with a volume of 510 mL, hermetically closed with the OxiTop measuring head. A CO_2_ scavenger was added to sequestrate carbon dioxide produced during biodegradation. Biotic consumption of the oxygen present in the free volume of the system was measured as a function of the decrease in pressure.

Samples were tested in triplicate. Raw oxygen consumption data (mg O_2_/L) were corrected by subtracting the mean values of the blanks obtained by measuring the seawater’s oxygen consumption without any test material. After this subtraction, values were normalized on the mass of the individual samples and referred to 100 mg of material (mg O_2_/100 mg material). Finally, the means of the triplicates were calculated and plotted vs time.

### 2.14 Maintenance and Growth Condition of Corals

#### 2.14.1 Acclimatization Tank

The reef-building coral *Stylophora pistillata* was used as the test species. Experiments were performed with 132 fragments (∼2–6 cm in length) obtained from eight large mother colonies/genotypes raised inside tanks of the Genoa Aquarium (Genoa, Italy). The nubbins were randomly divided among the different experiments and groups. Before the experiment, fragments were kept under controlled conditions in an acclimatization 3100 L tank (photoperiod was 12 h:12 h light:dark) to recover for 1 month. Colonies were fed twice a week with a food mixture in a solution containing Tetraselmis algae and Rotifera to improve recovery. During the day, the tank was set up as a semi-open system, constantly supplied (about 300 L every hour) with previously filtered seawater (by UV filtration) pumped from a 50 m depth outside the Foranea dam of Genoa. The temperature was constantly set up at 25 °C. Corals were illuminated with an HQI lamp (400w 10,000K Nepturion BLV) at an average irradiance of 250 μmol photons m^–2^ s^–1^. At night, the tank was set up as a closed system, where the water circulation pump (Argonaut AV150-2DN-SB 220v) was set between 10 and 13 m^3^ per hour to ensure a complete change of water every 25 min. The water from the tank, once taken by the pump, passed through a filtration system consisting of a sand filter (0.4 mm, Astralpool ARTIC) and a UV filter (Panaque 750 s, with four 40 W lamps embedded); subsequently the water was reinserted into the tank. Moreover, 50 L of calcium hydroxide solution at a concentration of 18 g/L was added dropwise every night to facilitate the calcification of corals, enhancing their growth.

#### 2.14.2 Experimental Tank

After the acclimatization, corals were transferred to the experimental tanks. Two tanks were prepared with the same parameters. Specifically, during the day, the tanks (400 L) were set up as an open system that constantly receives filtered seawater from another water circulation system from the aquarium, supplied with 40 L per hour. Before entering the experimental tanks, the water passes through a filtration system consisting of a sand filter (0,4 mm, Astralpool ARTIC) that removes coarse particles, through an ultraviolet filter (WEDECO KATADYN AG), to sterilize the water, and finally through a protein skimmer (N Sguassero, Italy) that removes the organic agglomerates. Corals (photoperiod 12 h:12 h light:dark) were illuminated by two 96 W metal halide lamps (Sylvania, Domilux) at an irradiance of 250 μmol photons m^–2^ s^–1^. Furthermore, the tanks were supplied with 2 porous air stones for aquaria to keep the water constantly oxygenated and in motion and with an aquaria pump (EHEIM 2275-02-0, max flow: 1250 L/h) to have further biological filtration. At the end of each treatment, the tanks were completely emptied and filled with freshly filtered seawater. The experimental temperatures of 25, 29, and 33 °C were regulated by two aquarium heaters (NEWA Therm Eco 300w). The chemical and fiscal parameters of the water were regularly analyzed (before and after each treatment) and kept constant throughout the experiment, in both the acclimatization and experimental phases.

### 2.15 Application and Biocompatibility

Strips of Z/P 6/4, 5/5, 4/6, 3/7, 2/8, and 1/9 samples (1.5 × 9.0 cm) were applied on the nubbins of *S. pistillata* after the acclimatization to verify their applicability. One side of the strip was fixed on the support of the nubbin by using hot silicone glue. The rest of the strip was rolled around the coral, and PVP adhesive capacity in wet conditions helped in the initial attachment (see Figure 3A). For each Z/P ratio film, two nubbins were used for this experiment. The state of the materials and the nubbins was monitored for 24 h.

Likewise, strips of Z/P 6/4 and Parafilm tape were wrapped around the corals for a long-term biocompatibility assay. Parafilm was fixed at the support using a resin for aquaria because it melts in contact with the hot glue. It was used as a positive control of a general obstacle that can remain entrapped among the branches of the corals. The health status of corals treated with Z/P 6/4 and Parafilm, and untreated corals was monitored for 15 days (*n* = 4). Photographs of the corals were taken after 1 h, 6 h, 24 h, 3 days, 9 days, and 15 days. Morphological aspects and biomolecular markers were chosen as parameters to define the viability of corals. Coral color, polyps’ opening, Chls, and enzymes were quantified. The coral color was evaluated by the color chart shown in Figure S4A; for the polyps’ opening, a different score was given to their condition, and the corresponding values are displayed in Figure S4A. Chls and enzyme values were provided by specific assays described in the following sessions.

### 2.16 Induced Thermal Stress

Corals were tested at 29 and 33 °C for 36 h to simulate a bleaching event. After the acclimatization, corals kept at 25 °C (*n* = 4) were also evaluated as the initial condition of the stress levels. Three experimental groups were assessed: untreated nubbins, nubbins treated with Z/P 6/4, and Z/P 6/4 without curcumin (Z/P 6/4 no curcumin). This last group was added to confirm that differences eventually present among the groups that occurred due to the curcumin. Nubbins of the experimental groups were removed from the tank after 6, 12, 24, and 36 h at 29 and 33 °C, transferred in dry ice, and then stored at −80 °C for further analyses. For each time point of each experimental group, four nubbins were used. At 25 °C, four nubbins for each group were sampled after 36 h.

### 2.17 Molecular Analysis

#### 2.17.1 Quantification of Chl *a* and *c2*

Coral tissue was blasted off from frozen fragments using airflow from a 1000 μL pipette tip connected via a rubber hose to a benchtop air pressure valve and 5 mL of ice-cold phosphate buffer saline.^46^ The tissue slurry was homogenized and centrifuged at 3600*g* for 4 min. The supernatant was removed, and the remaining pellet was incubated in 100% acetone for 24 h in the dark at 4 °C. Following extraction, the sample was re-centrifuged at 3600*g* for 4 min. The supernatant was used to determine concentrations of Chl *a* and *c2* from the fluorescence measured at 630, 663, and 750 nm, applied to dinoflagellate-specific equations,^47^ and normalized to the coral surface area.

The remaining skeletons of the coral fragments were soaked in 10% bleach and left to dry (48 h). The surface area of fragments was measured using the paraffin wax dipping method.^48^ The change in weight due to wax addition was compared against a standard curve of dipped clay cylinders of known surface area to calculate the skeletal surface area of each fragment.

#### 2.17.2 Quantification of SOD and CAT

##### 2.17.2.1 Protein Extraction

Coral fragments were mashed with a pre-chilled mortar and pestle and then transferred into tubes and homogenized in 750 μL of lysis buffer (Tris–HCl 50 mM, pH 7.4, NaCl 150 mM, glycerol 10%, NP40 detergent 1%, EDTA 5 mM), containing 1 mM phenylmethylsulfonylfluoride (Sigma-Aldrich). A first centrifugation step (5 min, 3000 rpm) was performed to remove skeletal components. Samples were then subjected to a second centrifugation step (15 min, 14,000 rpm, 4 °C), and the supernatant was sampled and frozen immediately (−80 °C) until subsequent assays. The total protein content of each sample was determined through the Bradford method using BSA as a reference to design a calibration curve.

##### 2.17.2.2 CAT Activity Assay

CAT activity was assessed by considering the peroxidation function of the enzyme. The method is based on the enzyme’s degradation of hydrogen peroxide (H_2_O_2_), as described in Bergmeyer and Graßl.^49^ The reaction solution (containing 50 mM sodium phosphate buffer pH 7.5, 12 mM H_2_O_2_) was mixed in a 1 mL cuvette with different sample volumes. The decrease of H_2_O_2_ was followed spectrophotometrically at 240 nm (Varian Cary 50 Scan spectrophotometer, Agilent Technologies). Results are expressed as units (U) of enzyme per mg of proteins, and in this case, U refers to *k*, the first-order kinetic constant (min^–1^), as previously described in ref (50).

##### 2.17.2.3 SOD Activity Assay

SOD activity was assessed according to Vance et al.^51^ As SOD competes with ferricytochrome c for oxygen radicals, its activity was detected as the ability to inhibit the reduction of ferricytochrome c by O^2–^ generated from the xanthine/xanthine oxidase system. For the reaction mix, the following reagents (purchased from Sigma-Aldrich), ferricytochrome c 0.01 mM, EDTA 0.1 mM, xanthine 0.01 mM, and xanthine oxidase 0.0061 U, were used in a final volume of 1 mL. Different volumes of each sample were tested and added to the reaction mix to determine the 50% inhibition of the reaction rate. The rate of reduction of ferricytochrome c was followed spectrophotometrically at 550 nm, 25 °C, through a Varian Cary 50 Scan Spectrophotometer (Agilent Technologies). Under the above conditions, one unit of SOD was defined as the amount of enzyme inhibiting the reduction of ferricytochrome c by 50%. Results are expressed as units (U) of enzyme per mg of proteins.

### 2.18 Statistics

Statistical analyses were performed using Statistical Package for Social Sciences (SPSS) software (IBM, version 28.0.11). The Shapiro–Wilk test was used to assess the normality of data. One-way analysis of variance (ANOVA) was conducted, followed by a Tukey’s pairwise comparison of means of normally distributed data. Means of non-normally distributed data were compared using the Kruskal–Wallis one-way ANOVA. *p*-values less than 0.05 were considered statistically significant.

## 3 Results

### 3.1 Characterization of Zein/PVP-Based Biocomposites

**Figure 1:**
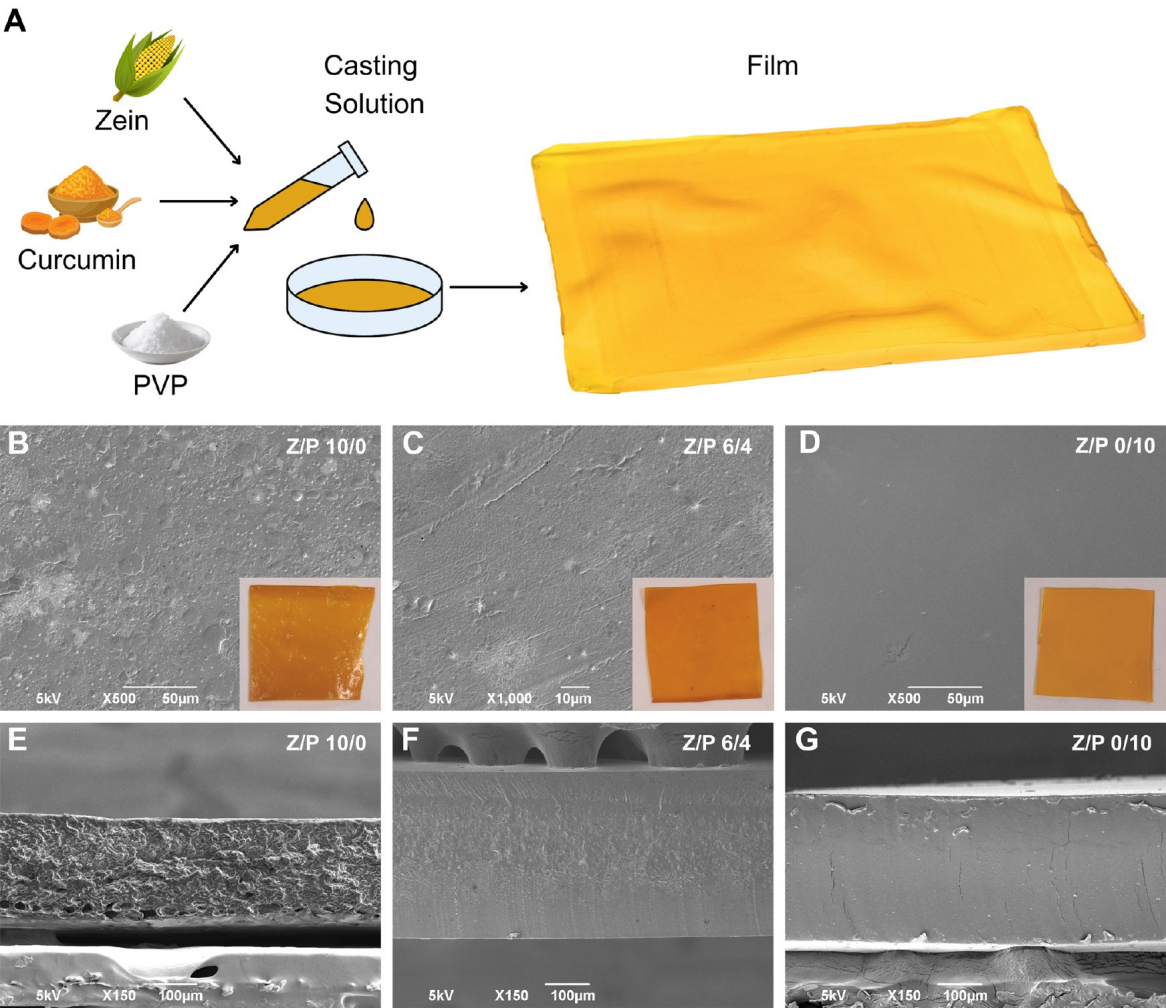
Morphology. (A) Representation of the film composition, preparation, and photograph of a Z/P 6/4 sample. (B, C, and D) Top-view SEM images of Z/P 10/0, 6/4, and 0/10 samples, respectively. (E, F, and G) Cross-section SEM images of Z/P 10/0, 6/4, and 0/10 samples, respectively.

Films were fabricated with 11 different zein/PVP (Z/P) weight ratios from 10/0 to 0/10. The films also included glycerol as a plasticizer and curcumin as an antioxidant agent. Labels and compositions of the produced samples are presented in Table S1. A schematic representation of the films’ components and a photograph of a plasticized film with a Z/P weight ratio of 6/4 and curcumin are shown in Figure 1A. The same quantities of curcumin and glycerol were loaded in all the films. Reference films without curcumin and films made of pristine components such as zein, PVP, glycerol, and a mix of them were also produced and utilized for comparison when required.

The Z/P biocomposites were colored orange due to the curcumin and had an average thickness of 250 ± 60 μm. Representative SEM images of the top view for Z/P 10/0, 6/4, and 0/10 samples are reported in Figure 1B, C, and D, respectively. The Z/P 10/0 samples showed irregular and rough surfaces, while Z/P 0/10 had a smooth surface, a typical feature of PVP films.^43^ Instead, Z/P 6/4 presented a combination of the two scenarios. The surface morphology also affected the transparency of the films, as can be noticed in the insets in Figure 1B–D. A similar trend was found when analyzing the cross-section images for Z/P 10/0, 6/4, and 0/10 samples reported in Figure 1E, F, and G, respectively. Indeed, the cross section for Z/P 0/10 was entirely smooth and compact, while the presence of zein made the cross section more irregular, as can be noticed for the Z/P 10/0 and Z/P 6/4 in Figure 1E and F, respectively.

**Figure 2:**
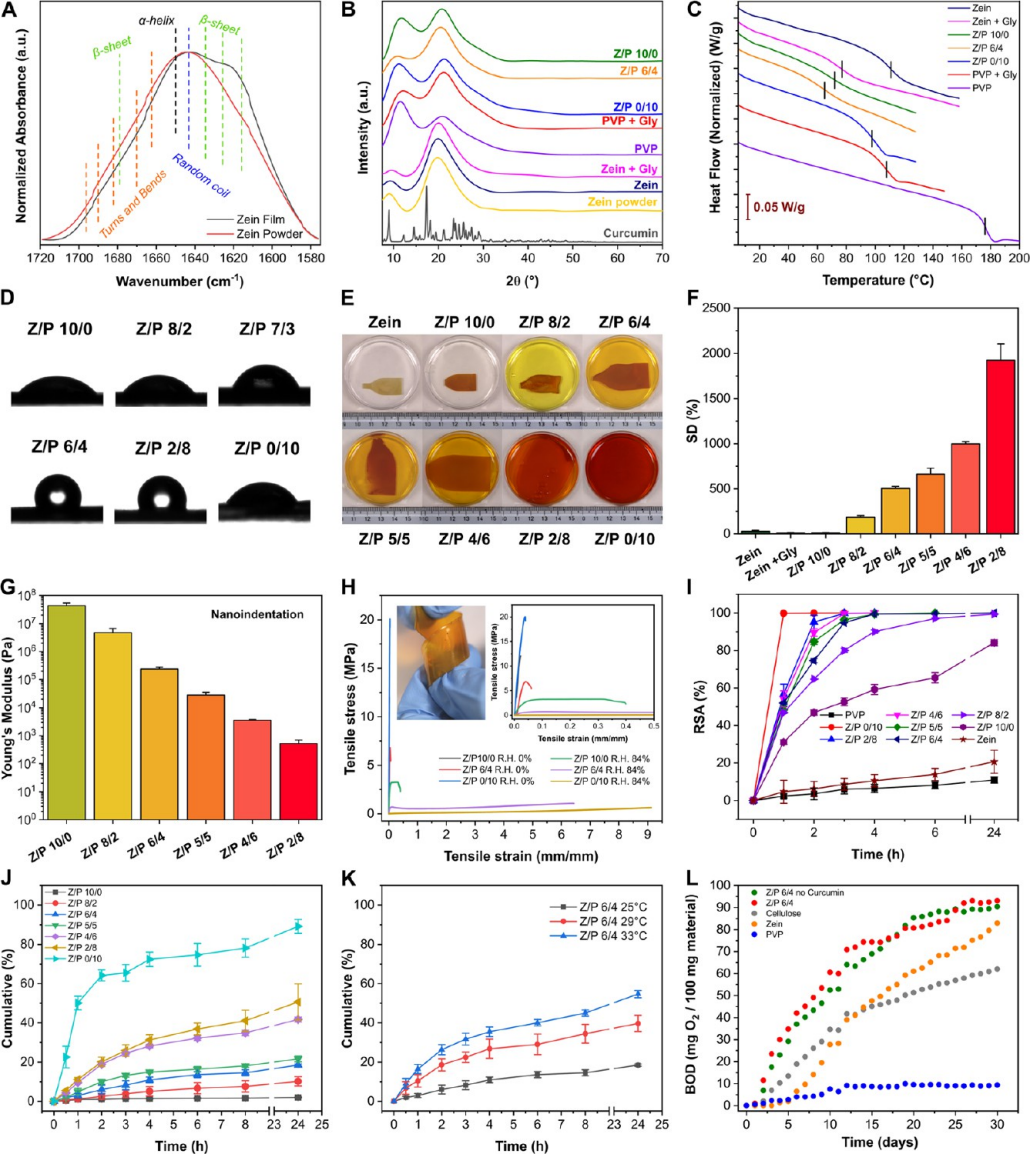
Characterization of the materials. (A) Normalized FTIR spectra of zein powder and zein film in the region of amide I. The attribution of the main conformational states of the protein are reported. (B) XRD patterns of zein powder, zein, zein + Gly, Z/P 10/0, Z/P 6/4, Z/P 0/10, PVP + Gly, and PVP samples. (C) DSC curves of the zein, zein + Gly, Z/P 10/0, Z/P 6/4, Z/P 0/10, PVP + Gly, and PVP samples in the range of temperatures between 8 and 200 °C. The glass-transition temperatures have been highlighted with black lines. (D) Photographs of the static water contact angle analysis for the Z/P 10/0, 8/2, 7/3, 6/4, 2/8, and 0/10 samples. (E) Photographs of zein, zein + Gly, Z/P 10/0, 8/2, 6/4, 5/5, 4/6, 2/8, and 0/10 samples after immersion for 24 h in seawater. (F) Swelling degree values for zein, zein + Gly, Z/P 10/0, 8/2, 6/4, 5/5, 4/6, and 2/8 samples after immersion for 24 h in seawater. (G) Results of the nanoindentation test for the Z/P 10/0, 8/2, 6/4, 5/5, 4/6, 2/8, and Parafilm samples after immersion for 24 h in seawater. (H) Stress–strain curves for the Z/P 10/0, 6/4, and 0/10 samples at 0 and 84% R.H. The insets show the following: on the right a zoom of the stress–strain curves for Z/P 10/0, 6/4, and 0/10 samples at 0 and 84% R.H in the strain range 0.0–0.5 mm/mm; on the left a representative photograph of a wrapped Z/P 6/4 strip. (I) Results of the ABTS scavenging assay for the zein, Z/P 10/0, 8/2, 6/4, 5/5, 4/6, 2/8, 0/10, and PVP samples. (J) Cumulative release of curcumin for the Z/P 10/0, 8/2, 6/4, 5/5, 4/6, 2/8, and 0/10 samples at 25 °C. (K) Comparison of the curcumin release from Z/P 6/4 at 25, 29, and 33 °C. (L) Biochemical oxygen consumption (mg O_2_/100 g material) as a function of time (days) for the zein, Z/P 6/4, Z/P 6/4 no curcumin, PVP, and cellulose samples.

A complete characterization of the physicochemical properties of the developed biocomposites is described in Figure 2. Films were chemically analyzed by ATR-FTIR. Zein powder was also evaluated for this analysis as a reference for the protein’s initial state. From up to down, the FTIR spectra of zein powder, zein film, zein + Gly, Z/P 10/0, Z/P 6/4, Z/P 0/10, PVP + Gly, PVP, and curcumin are displayed in Figure S1, and the main vibrational modes are highlighted. The salient changes among the spectra were found in the region of amide I (1720–1550 cm^–1^). This area is a source for obtaining information regarding proteins’ secondary structure and conformational state.^52^ Overlapped amide I bands of zein powder and zein film are shown in Figure 2A, and the colorful dash lines highlight the peaks of the conformational states. Turns and bends of zein were found at 1691,1677, 1668, and 1662 cm^–1^; the α-helix peak was present at 1654 cm^–1^; random coil contribution was at 1642 cm^–1^; and the typical β-sheet peaks were at 1638, 1629 and 1616 cm^–1^. Two more peaks were found at 1605 and 1594 cm^–1^ that were assigned to the aromatic C=C stretching due to zein’s high amount of aromatic amino acids. In the amide I area of the zein film sample, an evident increase in the contribution of the β-sheet peaks compared to the zein powder was observed. This variation was attributed to the fabrication procedure, where solvents and heat modify the protein conformation. Both can easily alter protein conformation and orientation in space.^53,54^ In the Z/P 6/4 spectrum, the β-sheet shoulder in the amide I area is still evident and is highlighted by the blue arrow in Figure S1. Noteworthy is that the C=O stretching of PVP appears between 1655 and 1640 cm^–1^, merging with the α-helix and random coil contributions, and making not reliable an investigation of the protein’s final state in the biocomposites using exclusively infrared spectroscopy.

XRD spectroscopy was used to define the physical state of the biocomposites, and the obtained patterns are reported in Figure 2B. PVP showed two broad peaks at 2θ 11.4 and 21.1°, typical of its amorphous state. Similarly, zein had two broad peaks centered at 2θ 9.3 and 19.9°, confirming its amorphous nature both in the form of powder and film. Instead, curcumin powder presented an XRD pattern with multiple narrow peaks indicative of a well-defined crystalline structure, whose peaks were confirmed by comparing with the reference pattern ICDD Card No. 00-066-1420. On the other hand, in the patterns of Z/P 10/0, 6/4, and 0/10 samples, two different peaks due to both zein and PVP were still visible while no peaks related to curcumin were observed, revealing that the antioxidant was incorporated in the amorphous structure of the polymeric matrices. This phenomenon occurs mainly because of PVP, which can disturb the intramolecular interactions of drugs, destroying their crystallinity.^55,56^

DSC was performed to define the *T*_g_ of the Z/P samples, and the main results are reported in Figure 2C. Before the DSC measurements, TGA was carried out to define the initial degradation point for each sample and the results are reported in Figure S2A,B. *T*_g_ for the zein film was at ≈112 °C. The introduction of glycerol in the Zein + Gly sample decreased *T*_g_ to 77 °C, a typical plasticizing effect. Likewise, in the Z/P 10/0 film, which includes both the plasticizer and the antioxidant, *T*_g_ occurred at 72 °C, suggesting that curcumin also played a role in the reduction of *T*_g_. Pure PVP had a *T*_g_ at ≈176 °C. Also in this case, both glycerol and curcumin affected the *T*_g_ of PVP. Indeed, the *T*_g_ for the PVP + Gly sample was at 108 °C while that for Z/P 0/10 was at 98 °C. Finally, in Z/P 6/4, *T*_g_ decreased at 65 °C, supporting the possibility of a single phase. A similar behavior has already been described by Sionkowska et al.^57^ when PVP and the protein collagen were combined. Here, a unique *T*_g_ was found and confirmed a perfect blend and strong interactions between the PVP and the protein. In addition, the decrease of *T*_g_ due to the presence of curcumin in PVP was also previously observed and explained a strong interaction between the two components.^58^

Biocomposites’ surface and bulk properties in response to the interaction with water were investigated by WCA and swelling tests. Values of WCA for the Z/P samples are reported in Table S2, whereas photographs of water drops deposited on Z/P 10/0, 8/2, 7/3, 6/4, 2/8, and 0/10 are shown in Figure 2D. Variations of WCA were linked with the quantity of zein and PVP in the polymeric matrices. Specifically, WCA values between 45 and 60°, indicating hydrophilic samples, were observed for the samples of pristine zein, zein + Gly, Z/P 10/0, 9/1, 8/2, and 7/3. Below this threshold of zein fraction in the samples, the WCA increased drastically with values over 100° for the Z/P ratios from 6/4 to 1/9, showing an inversion of the surface into hydrophobic. Z/P 0/10, PVP + Gly, and pure PVP had a WCA of 56, 60, and 61°, respectively, returning to fully hydrophilic surfaces.

Photographs and SD for the films immersed in seawater for 24 h are reported in Figure 2E and F, respectively. Zein, zein + Gly, and Z/P 10/0 showed SDs between 10 and 20%, highlighting the poor swelling properties of zein and suggesting that curcumin did not affect this property. Conversely, the overall SD increased when PVP was present in the matrix. In fact, Z/P 8/2 showed an SD of ≈185% while Z/P 6/4, 5/5, 4/6, and 2/8 had an SD of ≈500, 660, 1000, and 1925%, respectively. Samples with a higher weight ratio Z/P, thus 1/9 and 0/10 films, dissolved after 24 h; therefore, no values of SD could be calculated. Furthermore, the morphology of swollen Z/P 6/4 was investigated by SEM, and the top-view and cross-section images are reported in Figure S3A and B, respectively. The overall thickness increased by 40% (350 ± 30 μm), and pores appeared on the surface and in bulk.

Mechanical properties for the Z/P 10/0, 8/2, 6/4, 5/5, 4/6, and 2/8 samples were evaluated in the swollen state after immersion for 24 h in seawater, and the results are reported in Figure 2G. Z/P 10/0 showed the highest YM value of ≈43.4 ± 10.4 MPa, while the YM values were 4.7 ± 2.0 MPa for Z/P 8/2, 239.5 ± 36.4 kPa for Z/P 6/4, 27.9 ± 7.3 kPa for 5/5, 3.5 ± 0.2 kPa for 4/6, and only ≈526.3 ± 167.8 Pa for Z/P 2/8. The Z/P 9/1 and 0/10 samples cannot be considered due to their dissolution within 24 h after water immersion.

Additionally, the mechanical properties of the films at 0 and 84% R.H. are reported in Table S2, and stress–strain curves for the Z/P 10/0, 6/4, and 0/10 samples at 0 and 84% R.H. are shown in Figure 2H. At dry conditions (0% R.H.), pristine PVP had a Young’s modulus (YM) of ≈1042 MPa, tensile stress at maximum load (TSML) of ≈29 MPa, and elongation at break (EB) of 3.8%. A similar behavior was observed for the pristine zein with YM of ≈790 MPa, TMSL of 8.5 MPa, and EB of 1.4%. For the samples PVP + Gly, zein + Gly, Z/P 0/10, and Z/P 10/0, an important reduction of these values compared to the pristine polymers was noticed due to the plasticizer effect of glycerol. The combination of the two polymers led to a further slight reduction of the YM and TSML and a slight increase of the EB. Indeed, the combination Z/P 6/4, Z/P 5/5, and Z/P 4/6 showed YM values of ≈356, 350, and 380 MPa; TMSL of ≈5.6, 6.2, and 9.4 MPa; and EB of ≈4.2, 4.7, and 5.5%, respectively.

As can be noticed in Figure 2H, at 84% R.H., Z/P 10/0 and ZP 0/10 presented different stress–strain curves compared to the 0% R.H. curves previously described due to the humidity that acted as a plasticizer. Indeed, the registered values of YM, TSML, and EB for Z/P samples underwent substantial variations. Zein became more ductile and had YM, TSML, and EB of ≈147 MPa, 3.8 MPa, and 6%, respectively. Reduction of the YM and TSML and increase of the EB compared to the dry condition were also observed for the samples zein + Gly and Z/P 10/0. Instead, Z/P 9/1, 8/2, and 7/3 showed a slighter inversion of the trend, having values of YM and TSML higher than the samples with only zein. This inversion of the behavior was also noticed for the same samples in the dry tests, suggesting a different rearrangement of the polymeric matrices when a small percentage of PVP was present in the system. From the Z/P ratio 6/4, the plasticizing effect induced by moisture due to the presence of PVP started being more evident. This latter showed a YM of ≈ 25 MPa, TSML of 1.1 MPa, and EB of 504%. Further increase of the PVP percentage inside the biocomposite matrices led to values of YM and TSML of ≈0.3 and 0.5 MPa, respectively, and EB of more than 900%. This means that by regulating the Z/P content inside the matrices and the humidity, the biocomposites can behave as brittle, ductile, or elastomeric materials, potentially allowing their use with different types of corals.

The antioxidant capacity of biocomposites was verified by the ABTS scavenging assay, and the main results are shown in Figure 2I. The produced biocomposites loaded with curcumin displayed strong antioxidant activity after 24 h. However, their action was affected by their physicochemical features and release profile. Z/P 0/10 scavenged all the free radicals in 1 h due to its fast dissolution. By increasing the zein content, the release profile was delayed, as previously shown, and consequently, the scavenging activity was also delayed. Indeed, Z/P 10/0 resulted in being the slowest scavenger, having an RSA value of 85% at the end of the assay. As expected, pure zein and PVP films did not show a significant capacity to block the free radicals.

However, curcumin should be released from the Z/P samples after their immersion in water to exploit its beneficial properties. For this reason, release tests at 25 °C were carried out; the findings are reported in Figure 2J. Z/P 0/10 had the fastest release profiles, with ≈75% of curcumin diffused in the first 4 h. Instead, the Z/P 2/8 and 4/6 samples released 50 and 40%, respectively, of the incorporated curcumin after 24 h. A further increase of the zein quantity inside the biocomposite matrix induced a slow and controlled release profile of curcumin in water, reaching a total release of less than 20% of the curcumin incorporated in these samples.

The release and diffusion of a drug from a polymeric matrix within the water are strongly affected by temperature.^59,60^ The curcumin release profile from the Z/P 6/4 sample was analyzed at 29 and 33 °C and compared with the profile at 25 °C (see Figure 2K). Interestingly, after 24 h, 39 and 55% of the loaded curcumin were released at 29 and 33 °C, respectively. These values are significantly higher than 18% of curcumin diffused at 25 °C. Notably, these temperatures were used as reference temperatures for the coral heat stress experiments presented next.

Finally, the BOD and consequent degradation level for PVP, zein, Z/P 6/4 no curcumin (a reference sample without curcumin), and Z/P 6/4 films were studied for 30 days. Cellulose was tested as a positive control of biodegradation. The main results are reported in Figure 2L. PVP films showed the slowest degradation rate, obtaining a final value of 9 mg O_2_/100 mg material. On the other hand, cellulose consumed 63 mg of O_2_/100 mg of material. Interestingly, pure zein, Z/P 6/4 no curcumin, and Z/P 6/4 samples showed values of 83, 90, and 93 mg O_2_/100 mg material, respectively, demonstrating to be even more biodegradable in seawater compared to cellulose. Notably, curcumin did not affect, either positively or negatively, the samples’ biodegradability.

### 3.2 Application and Biocompatibility in Corals

**Figure 3:**
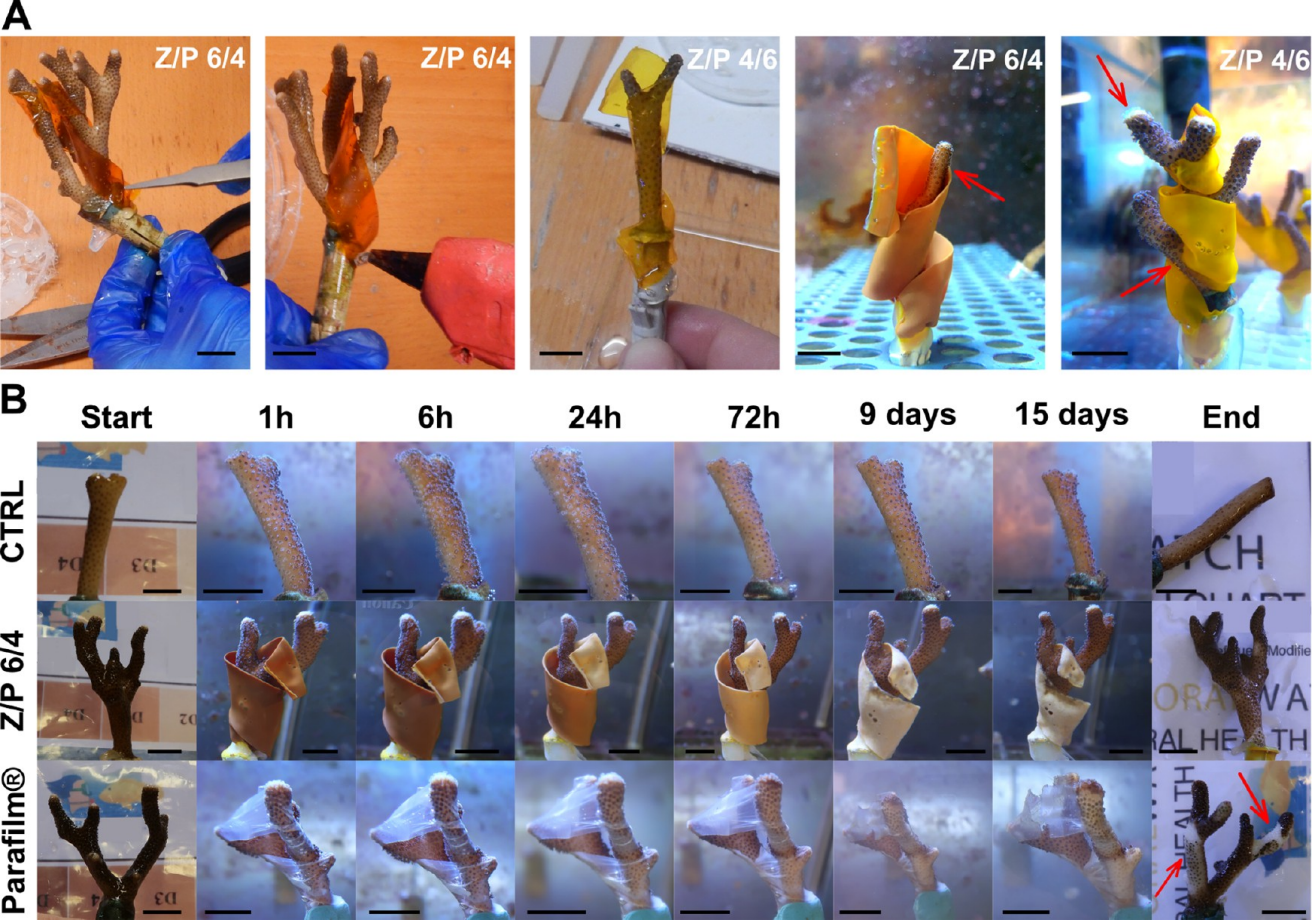
Application of the materials and biocompatibility tests. (A) Photographs of the application modality of strips of the Z/P biocomposites. (B) Photographs of the nubbin control, treated with Z/P 6/4 and with Parafilm for 15 days. The black bars indicate 1.2 cm.

*Stylophora pistillata* was used as a coral model for these experiments. The biocomposites were applied by fixing the strip on the nubbin support using a hot-glue gun. Also, the adhesive properties of PVP in wet conditions^30^ helped to stabilize the initial adhesion to the coral surface. After some minutes in the water tank, the strips started swelling and became like a soft “scarf” wrapped around the coral at a distance between 0.5 and 1 cm, as displayed in Figure 3A. Z/P 6/4, 5/5, 4/6, 3/7, 2/8, and 1/9 were tested on coral fragments for 24 h (see Figure S4). The Z/P 1/9 sample was fully dissolved, and the Z/P 2/8 film was partially degraded. Instead, Z/P 3/7, 4/6, 5/5, and 6/4 did not undergo macroscopic variations. All the biocomposites did not affect the viability of the corals in this timescale. Subsequently, a long-lasting “biocompatibility” test was performed comparing corals treated with the Z/P 6/4 biocomposite, treated with Parafilm, and untreated, and the photographs of the experiment are reported in Figure 3B. Parafilm is a control for simulating a material that can remain blocked among the corals’ branches and affect the colony’s health. After 15 days, the coral wrapped with the Z/P 6/4 material did not show any relevant macroscopic changes in its morphology, such as color and polyp’s opening (see Figure S5A,B), and its tissue condition compared to the control. On the other hand, necrosis and paling signals left by Parafilm can be noticed in the coral wrapped with this material, and the red arrows in Figure 3B highlight them.

The mechanical and WVP properties for Z/P 6/4 and Parafilm were compared in Figure S6A,B to investigate the reasons for this outcome. Nanoindentation measurement on Parafilm showed a YM of ≈66 ± 27 MPa, a value higher than that of the Z/P 6/4 sample (YM = 239.5 ± 36.4 kPa). Noteworthy is that Parafilm was more rigid even than the other Z/P samples, where the highest YM value was 43.4 ± 10.4 MPa for the Z/P 10/0 sample. The calculated WVP and WVTR values for Z/P 6/4 were 2930 ± 292 g/m^2^·day and 2.89·10–4 ± 1.14·10–5 g/m·day·Pa, respectively, while Parafilm did not allow any diffusion of water through its matrix.

### 3.3 Bleaching Mitigation in Induced Heat-Stress Conditions

We have chosen the Z/P 6/4 films to treat the corals under heat stress conditions to evaluate their effectiveness in mitigating the bleaching, due to their optimal properties that are analyzed in the Discussion section. Corals untreated and treated with Z/P 6/4 films were placed in tanks at 25, 29, and 33 °C for 36 h to investigate the coral response during the induced condition of heating stress. For these experiments, films of Z/P 6/4 without curcumin were also tested as a control for evaluating the efficacy of the antioxidant compound. Morphological (color score and polyps state) and physiological (Chls and enzymes) parameters were monitored to evaluate the overall status of corals. The visual appearance of the untreated control corals and corals treated with Z/P 6/4 no curcumin and with Z/P 6/4 at 25 °C is reported in Figure S7A; the color score and the polyp monitoring results are shown in Figure S5C. At the same time, the concentrations of chlorophylls ([Chls]) and the activity of antioxidant enzymes are displayed in Figures S7B and C, respectively. After 36 h at 25 °C, no significant changes were observed between the start and the end of the experiment. Observations show that both the films with and without the curcumin did not affect the physiological parameters and overall integrity of the coral colony. The quantification of Chls in samples provides a quantitative estimate of coral bleaching and health. No significant differences in concentrations of Chls *a* and *c2* ([Chl *a*]; [Chl *c2*]) were noted between the different treatments. Similarly, enzyme activities between 0.1 and 0.2 U/mg of SOD and in the range of 2–5 K/mg of CAT were found.

**Figure 4:**
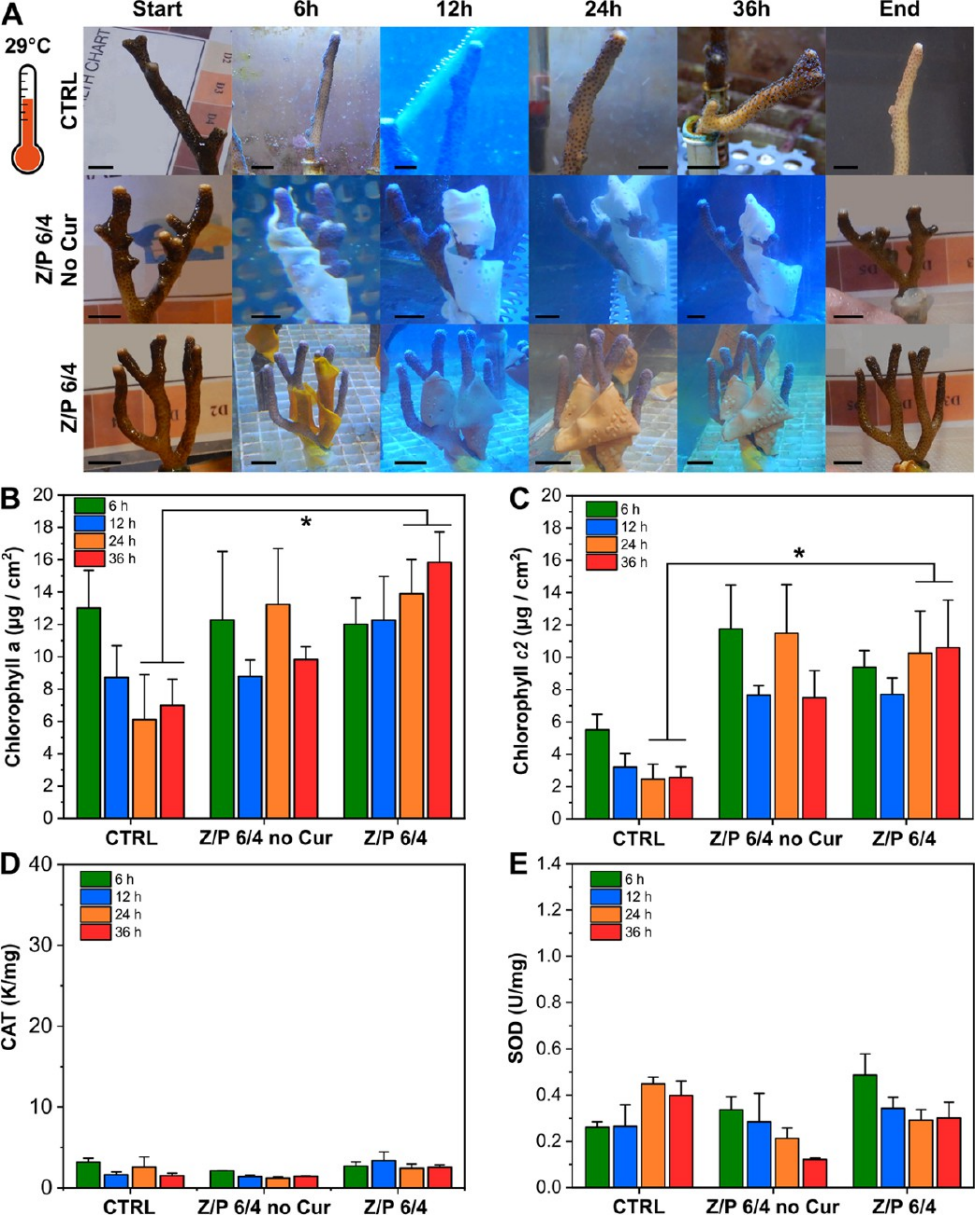
Test at 29 °C. (A) Photographs of untreated nubbin as well as those treated with Z/P 6/4 without curcumin and with Z/P 6/4 at 29 °C for 36 h. The black bars indicate 1.2 cm. (B) Concentration of chlorophyll *a* for the control, Z/P 6/4 no curcumin, Z/P 6/4 samples at 29 °C. *p < 0.05 Z/P 6/4 vs CTRL (24 h *P* = 0.012; 36 h *P* = 0.0001). (C) Concentrations of chlorophyll *c2* for the control, Z/P 6/4 no curcumin, and Z/P 6/4 samples at 29 °C. *p < 0.05 Z/P 6/4 vs CTRL (24 h *P* = 0.0001; 36 h *P* = 0.0001). (D, E) Activities of CAT and SOD, respectively, for the control, Z/P 6/4 no curcumin, Z/P 6/4 samples at 29 °C.

At 29 °C, a slight loss of color and reduction of the polyp’s opening were recorded for control samples. A reduction of the polyp’s opening was also noticed for the corals tested with Z/P 6/4 no curcumin, although no evident change of color score was observed. The corals wrapped with the Z/P 6/4 films did not show any visual sign of stress (see photographs and graphs in Figure 4 and Figure S5D). In the untreated control samples, [Chl *a*] decreased significantly with time (from 13 to 7 μg/cm^2^ after 36 h). Likewise, [Chl *a*] was reduced in nubbins treated with Z/P 6/4 no curcumin, while this did not occur in nubbins treated with Z/P 6/4.

A similar trend was observed for Chl *c2*. The [Chl *c2*] in untreated nubbins decreased during the experiment, while the Chl c2 in nubbins treated with the biocomposites were significantly stable over time (*p* values are reported in the capture of Figure 4). The [Chl *a*] and [Chl *c2*] in the corals at 29 °C are reported in Figure 4B and C, respectively.

**Figure 5:**
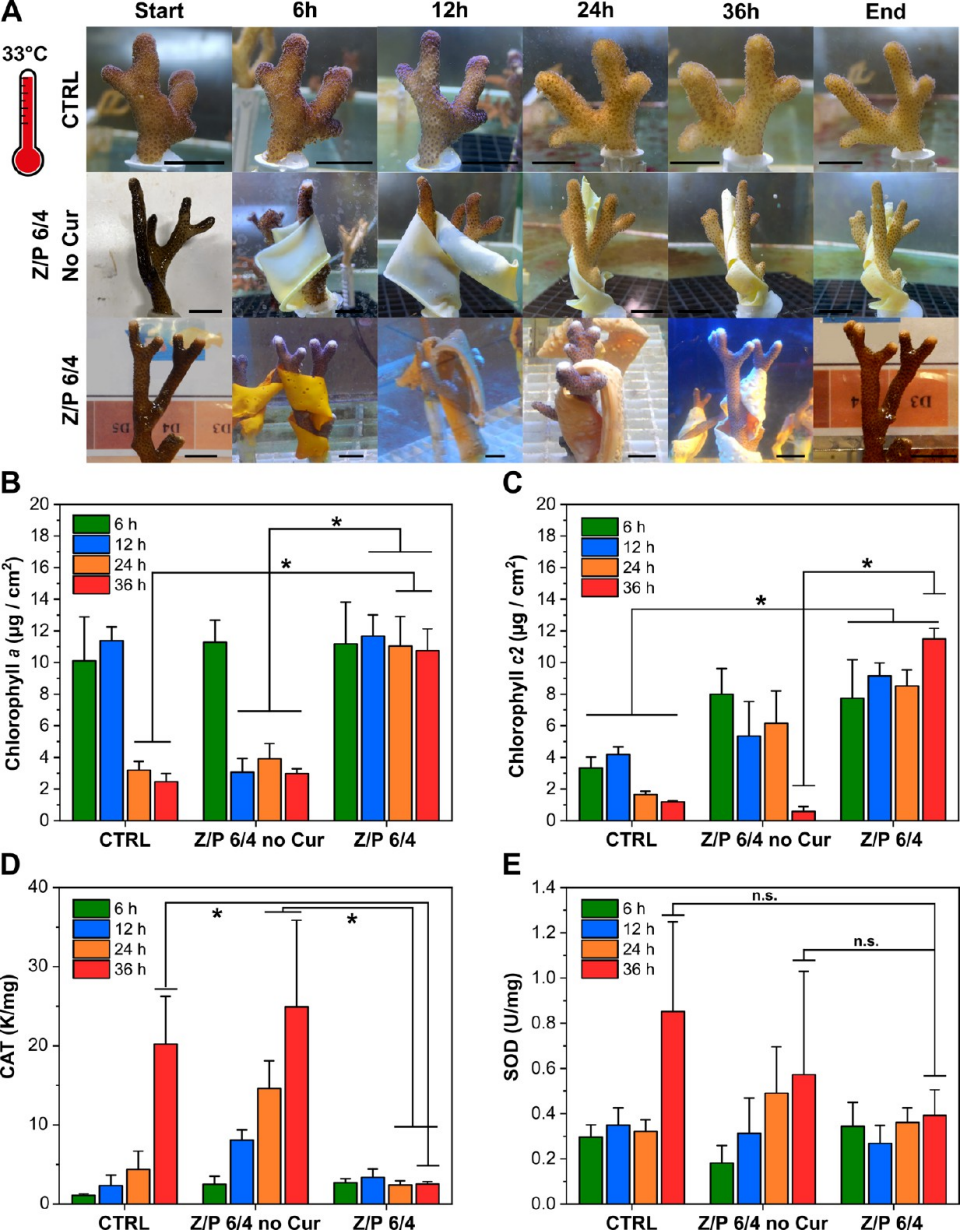
Test at 33 °C. (A) Photographs of untreated nubbin as well as those treated with Z/P 6/4 without curcumin and with Z/P 6/4 at 33 °C for 36 h. The black bars indicate 1.2 cm. (B) Concentration of chlorophyll *a* for the control, Z/P 6/4 no curcumin, and Z/P 6/4 samples at 25 °C. *p < 0.05 Z/P 6/4 vs CTRL (24 h *P* = 0.033; 36 h *P* = 0.008) or vs Z/P 6/4 no curcumin (12 h *P* = 0.007; 24 h *P* = 0.0001; 36 h *P* = 0.026). (C) Concentrations of chlorophyll *c2* for the control, Z/P 6/4 no curcumin, and Z/P 6/4 samples at 25 °C. *p < 0.05 Z/P 6/4 vs CTRL (6 h *P* = 0.003; 12 h *P* = 0.001; 24 h *P* = 0.011; 36 h *P* = 0.0001) or vs Z/P 6/4 no curcumin (36 h *P* = 0.0001). (D) Activity of CAT, respectively, for the control, Z/P 6/4 no curcumin, and Z/P 6/4 samples at 33 °C. *p < 0.05 Z/P 6/4 vs CTRL (36 h *P* = 0.006) or Z/P 6/4 no curcumin (24 h *P* = 0.002; 36 h *P* = 0.035). (E) Activity of SOD for the control, Z/P 6/4 no curcumin, and Z/P 6/4 samples at 33 °C.

CAT activity remained comparable to the values found at 25 °C (2–5 K/mg), while SOD showed an average increase for all the samples, reaching values of ≈0.3–0.5 U/mg, suggesting an initial response of the corals to the occurring thermal stress. The activities of CAT and SOD in control corals and corals treated with Z/P 6/4 no curcumin and Z/P 6/4 are reported in Figure 5D and E, respectively.

Finally, at 33 °C, control corals experienced an intense bleaching status, leading to a total loss of the color and closure of the polyps (Figure 5A and Figure S5E). [Chl *a*] decreased significantly from ∼10 μg/cm^2^ at the beginning of the experiment to 2 μg/cm^2^ (Figure 5B). The same trend was observed for [Chl *c2*] (Figure 5C). On the other hand, the activity of CAT and SOD intensively increased, reaching values of ≈20 K/mg and ≈0.9 U/mg, respectively (Figure 5D and E). The same, in those parameters, was observed for the nubbins treated with Z/P 6/4 no curcumin. Here, [Chl *a*] and [Chl *c2*] were significantly reduced after 36 h and the enzymes highly increased (*p* values are reported in the capture of Figure 5). By contrast, an opposite situation was found for the nubbins treated with Z/P 6/4. [Chl *a*] and [Chl *c2*] did not decrease but remained stable despite the thermal stress. The [Chl *a*] and [Chl *c2*] in nubbins treated with Z/P 6/4 were comparable to concentrations in the nubbins at 25 (no stress) and 29 (mild stress) °C. The activity of antioxidant enzymes did not show any statistically significant modulation, remaining in the same range of the 29 °C condition.

## 4 Discussion

Coral bleaching is a significant issue for coral reefs. So far, a few remediation techniques have attempted to reduce light exposure and seawater temperature or use probiotics but have failed in stemming this phenomenon. Projections about coral bleaching show that in the next years, a worrying increase in the frequency and severity of these events will occur,^7,61^ urgently calling for novel and more effective mitigation tools. This study presents a pioneering approach that uses biocomposite materials to bring molecules active in mitigating coral bleaching during thermal stress events. To achieve this aim, we designed underwater drug delivery systems for corals as free-standing films based on biodegradable biopolymers loaded with natural antioxidant curcumin and fabricated by solvent casting. Exploiting the natural origin and hydrophobicity of zein combined with the affinity of PVP for water, the produced biocomposites offered several features that make them ideal for use in aquaria, in-field rehabilitation strategies, and other scenarios in the oceans. By varying the zein/PVP weight ratio, we demonstrated that the biocomposites had different behaviors regarding water–material interactions and resistance, swelling and mechanical properties, and capacity to control the curcumin release. Chemical analyses highlighted that a change in zein conformation during the solvent-casting process occurred. Specifically, an increase in β-sheet content was observed. Instead, curcumin lost its crystallinity structure in the amorphous zein/PVP matrices, increasing its dispersion and overall bioavailability. Moreover, in the Z/P 6/4 biocomposite, a significant reduction of *T*_g_ for the single components zein and PVP was recorded. Taking these observations together, we can deduce that the biopolymers are very well-mixed and the curcumin is nicely dispersed and encapsulated into the composite polymeric matrix. On the other hand, when zein and PVP are combined at different weight ratios, zein undergoes rearrangements in the bulk at the supramolecular scale, as schematized in Figure S8. In detail, in the film with high zein content, the protein’s hydrophobic segments are internalized within the matrix, and the hydrophilic ones are more exposed externally together with PVP, as confirmed by the low contact angles of these samples. Reducing the zein content below the weight ratio Z/P 7/3, an inversion of this arrangement was found and more hydrophobic segments were exposed to the surface. In contrast, the hydrophilic ones are internalized in the matrix and strongly interact with the PVP. This phenomenon produced contact angles above 100° even in the samples with high PVP content. In addition, it ameliorated biocomposites’ water resistance and absorption, leading to the formation of soft hydrogels after immersion in seawater. Likewise, biocomposites’ mechanical properties ranged from stiffer to ductile materials, tunable based on Z/P weight ratio and moisture or water content. Also, the curcumin diffusion rate can be set, adjusted, and controlled by changing the Z/P ratio. Noteworthy is that despite its low solubility, curcumin was easily capable of diffusing due to the loss of its crystallinity, the high degree of dispersion inside the polymeric matrix, and the controllable swelling/erosion phenomena of the zein/PVP system. Studying the antioxidant release profile of Z/P 6/4, we also demonstrated an on-demand diffusion of curcumin as a function of temperature. Another crucial feature of the proposed biocomposites was their biodegradability. BOD analysis showed a high degree of biodegradability for the Z/P 6/4 with and without curcumin samples that immediately started their degradation in seawater, even earlier than pristine zein. This difference in the biodegradation profile can be explained by the simultaneous presence of zein, which, being a protein, is easily recognized by the bacteria, and PVP, which induces swelling of the material, allowing the entrance of water and the formation of pores as shown in Figure S3. Consequently, bacteria can quickly enter inside the bulk and speed up biodegradation.^62,63^ This outcome is even more evident in the first days of the test, where both Z/P 6/4 no curcumin and Z/P 6/4 samples started their degradation after only 2 days while the pristine zein in 6 days.

The coral *Stylophora pistillata* is an ecologically important reef-builder, forming a complex habitat for various marine species such as crabs, fishes, and various cryptic organisms.^64^*S. pistillata* is abundant in the lagoons, reef flats, and fore reefs^65^ and is widely distributed across the Indo-Pacific area.^64^ This hard-branching coral is usually defined as a “rat lab” as it has been used in numerous research works, specifically for studying the coral bleaching process.^66,67^

The biocomposites were tested using this coral to determine the method of application, the biocompatibility, and their efficacy in preventing bleaching. Strips of the biocomposites were wrapped around *S. pistillata* nubbins, whose branching morphology made anchoring the materials easy. This approach ensured close contact between the coral and the curcumin and optimized the in situ antioxidant concentration during the experiments. However, wrapping films around nubbins could threaten the normal physiology of coral. In nature, corals release mucus to trap and release foreign bodies such as sand and debris.^68−70^ Failure to remove the foreign body can affect the health of the colony. What we found was that the soft hydrogel nature of Z/P 6/4 and its capacity not to occlude the coral colonies were crucial points for ensuring a safe application. On the contrary, other types of material, such as Parafilm, a typical flexible and extendable waxy film used for bottle sealing, may affect the health and integrity of the coral colonies by causing tissue loss due to their rigidity and barrier effect if applied similarly. This interesting outcome can be a pillar of the future design of new biomaterials to deliver drugs to corals, suggesting how tuning mechanical and barrier properties can improve their efficacy and applicability on these organisms.

Regarding the bleaching experiments, the different temperatures (29 and 33 °C) and the final time point (36 h) were selected based on previous bleaching studies^11,12^ where these conditions were significantly triggering the bleaching in corals both from the macroscopic and biomolecular points of views. 25 °C is the physiological temperature for *S. pistillata* in aquaria.

The reported results demonstrated that the nubbins treated with curcumin showed higher tolerance to the coral bleaching at 33 °C without losing Chls and, consequentially, the zooxanthellae along the experiment time, as well as displayed enzymatic activities comparable to the condition at 29 °C. Diffusing from the films to the nubbins, curcumin appears capable of helping the coral in reducing the damages induced by heat stress. The coloration and Chls inside the nubbins are an indirect indication of the positive effects promoted by curcumin. Also, the lower activity of enzymes describes an overall improved condition of the corals during the thermal stress event. These outcomes suggest that antioxidant molecules can be new, natural, and efficient therapeutic agents for treating coral bleaching.

Similar effects have been found in human cells and animal models, where curcumin diffusing from a formulation reduces oxidative stress and the general inflammation condition.^40,41^ The biodegradable zein/PVP-based biocomposites provide an eco-friendly and biocompatible solution for delivering curcumin to corals underwater. The presented biocomposites can provide a versatile platform for various scenarios by customizing the material performance, moving from a high-content PVP to a high zein content inside the material. For instance, in the case of ongoing coral bleaching events, films made of high PVP content can be employed as a first-aid tool for quickly releasing high quantities of antioxidants. Alternatively, placing films mainly composed of a high zein content among the coral colonies can serve as a reservoir of natural antioxidants with a slow and controlled diffusion. In addition, the high biodegradability of these biocomposites gives the possibility to apply and leave many films underwater or eventually make a local and ad hoc treatment of the colony under stress.

The present study shows the mitigation effect of the biocomposites loaded with curcumin at one concentration only on *S. pistillata*; however, other concentrations of curcumin, as well as other antioxidants, such as ascorbic acid, hydroxycinnamic acids and derivatives, anthocyanins, and tocopherols, which could have a more substantial effect, should be evaluated. The biocomposites should also be tested in other coral species to investigate the universal use of this mitigation technique. The natural origin of the curcumin molecule does not potentially result in the problem of dispersing toxic molecules into the sea and consequently minimizes any potential environmental impact. Furthermore, the mechanism of action of curcumin during the coral bleaching should be investigated to determine if it acts by passively scavenging ROS/RNS or if it is also involved in the antioxidant responses cascade inside the coral cells and modulates the molecular pathways triggered after the heat stress. In addition, as previously described, different coral species have different susceptibilities to bleaching, and consequently, the biocomposites might require some adjustment to fit the need of specific species. For massive or encrusting coral growth forms, the application of film materials could be challenging, but antioxidants could be delivered using other platforms such as particles, fibers, or hydrogels. Therefore, in the perspective of a larger-scale application, further long-term analyses on the biomolecular background of the anti-bleaching effects, new tests on other coral species, and field applications during bleaching events are required.

## 5 Conclusions

In conclusion, we showed that zein/PVP-based biocomposites can potentially prevent coral bleaching. Recent advances in materials science, pharmaceutics, and biomaterials can be employed to project new methodologies and therapies to mitigate the current impacts on coral reefs and open a new and challenging frontier in drug delivery science. Moreover, antioxidants such as curcumin can be a powerful tool for tackling coral bleaching.

## Supporting Information Available

ATR-FTIR spectra of biocomposites; thermogravimetric analysis; SEM hydrogel Z/P 6/4; biocomposites on nubbins for 24 h; color and polyps’ opening; comparison Z/P 6/4 and Parafilm materials; test at 25 °C; zein/PVP assembly; composition of the produced samples; mechanical properties and water contact angle (PDF)
