# Supplementary material for: Biodegradable Zein-Based Biocomposite Films for Underwater Delivery of Curcumin Reduce Thermal Stress Effects in Corals
Source: ACS Appl Mater Interfaces. 2023 Jun 28;15(28):33916–31. doi: 10.1021/acsami.3c01166 (PMC10360034; doi:10.1021/acsami.3c01166)
Supplement: Supplementary file 1 — am3c01166_si_001.pdf [file am3c01166_si_001.pdf]

Supporting Information

Biodegradable Zein-Based Biocomposite Films for Underwater Delivery of Curcumin Reduce Thermal Stress Effects in Corals

Marco Contardi[a,b,], Marta Fadda[c], Valerio Isa[a,b], Yohan D. Louis[a,b], Andrea Madaschi[a,b], Sara Vencato[a,b], Enrico Montalbetti[a,b], Laura Bertolacci[c], Luca Ceseracciu[d], Davide Seveso[a,b], Silvia Lavorano[e], Paolo Galli[a,b,f], Athanassia Athanassiou[c,*], Simone Montano[a,b,*].*

[a]Department of Earth and Environmental Sciences (DISAT), University of Milan – Bicocca, Milan, 20126, Italy.

[b]MaRHE Center (Marine Research and High Education Center), Magoodhoo Island, Faafu Atoll, 12030, Republic of Maldives.

[c]Smart Materials, Istituto Italiano di Tecnologia, Genova, 16163, Italy.

[d]Materials Characterization Facility, Istituto Italiano di Tecnologia, Genova, 16163, Italy.

[e]Costa Edutainment SpA - Acquario di Genova, Genova, 16128, Italy

[f]Dubai Business School, University of Dubai, Dubai P.O. Box 14143, United Arab Emirates

*Corresponding authors: marco.contardi@unimib.it (M.C.); Athanassia.athanassiou@iit.it (A.A.); simone.montano@unimib.it (S.M.)

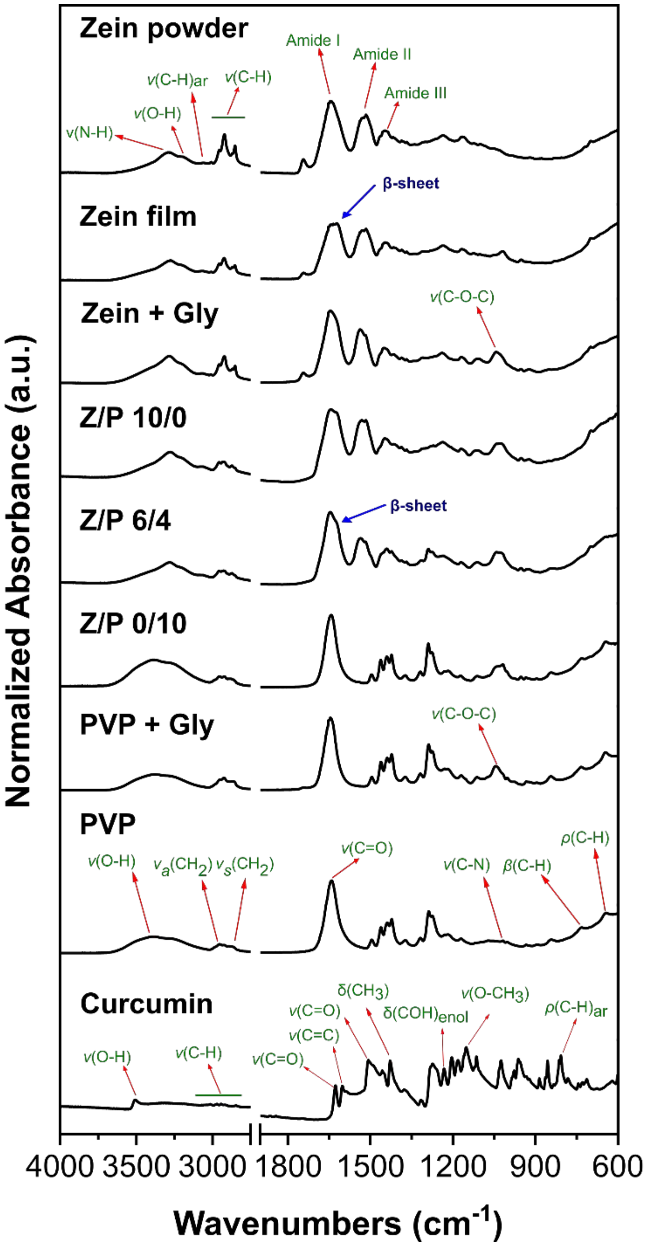

Figure S1. ATR-FTIR spectra. Infrared spectra for curcumin, PVP, PVP+Gly, Z/P 0/10, Z/P 6/4, Z/P 10/0, Zein + Gly, Zein film, and Zein powder (from bottom to up). The main vibration modes are shown.

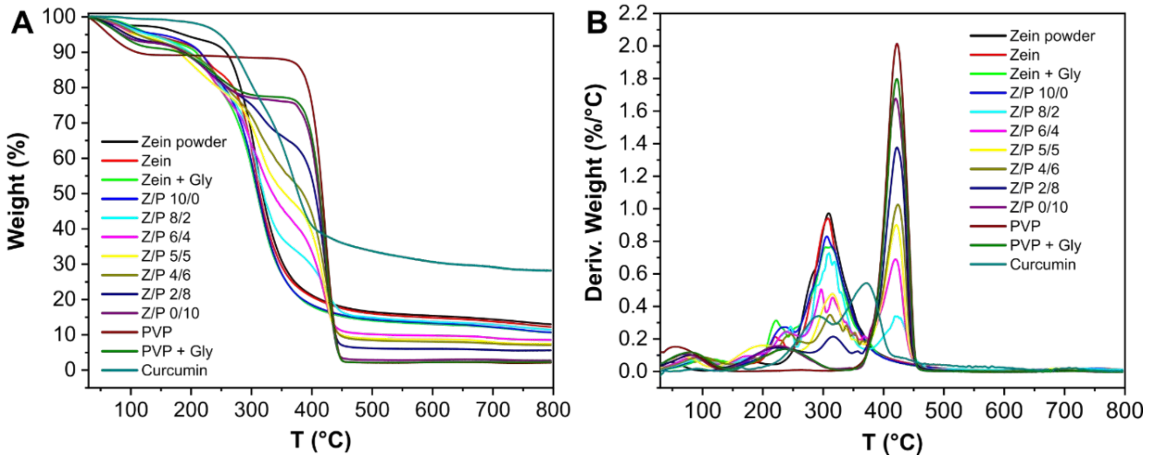

Figure S2. Thermogravimetric Analysis. A, B, TGA thermograms and derivative thermogravimetric curves, respectively, of the Zein powder, Zein, Zein + Gly, Z/P samples, PVP, PVP+Gly, and curcumin powder in the range of temperatures between 30 and 800 °C

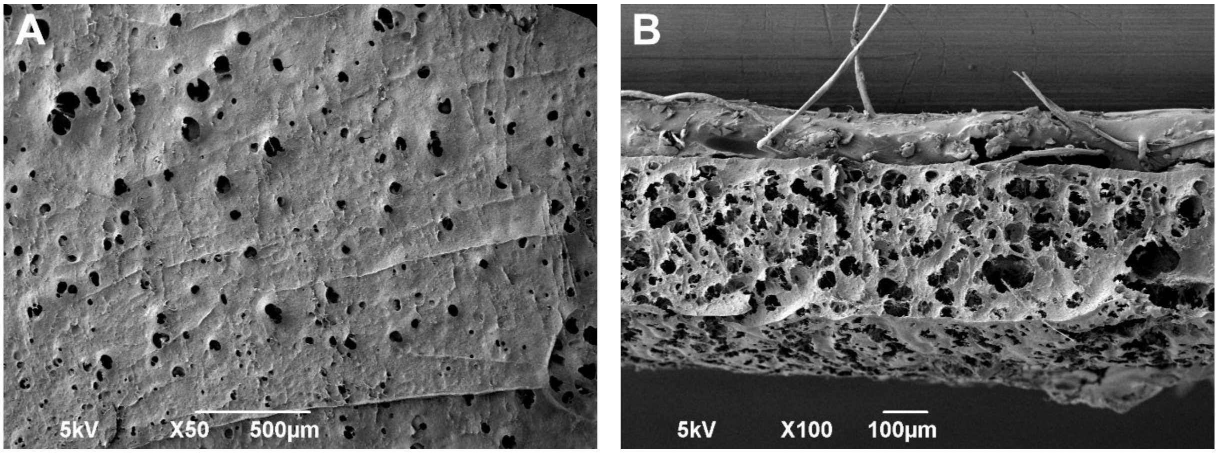

Figure S3. SEM Hydrogel A, B, SEM images of top-view and cross-section, respectively, for the Z/P 6/4 sample after 24 hours of immersion in water.

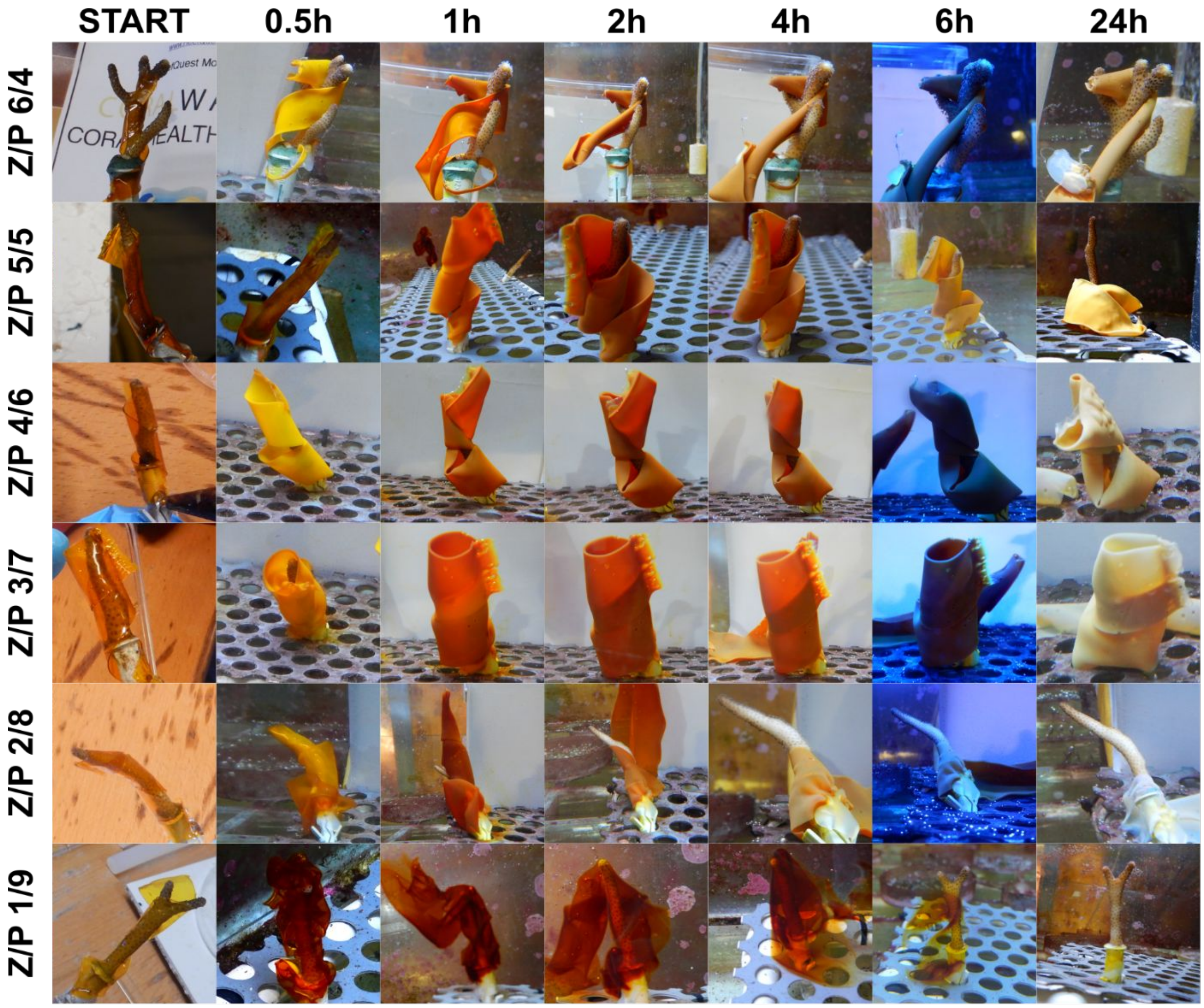

Figure S4. Biocomposites on nubbins. Testing underwater resistance in tank of Z/P 6/4, 5/5, 4/6, 3/7, 8/2, and 1/9 samples.

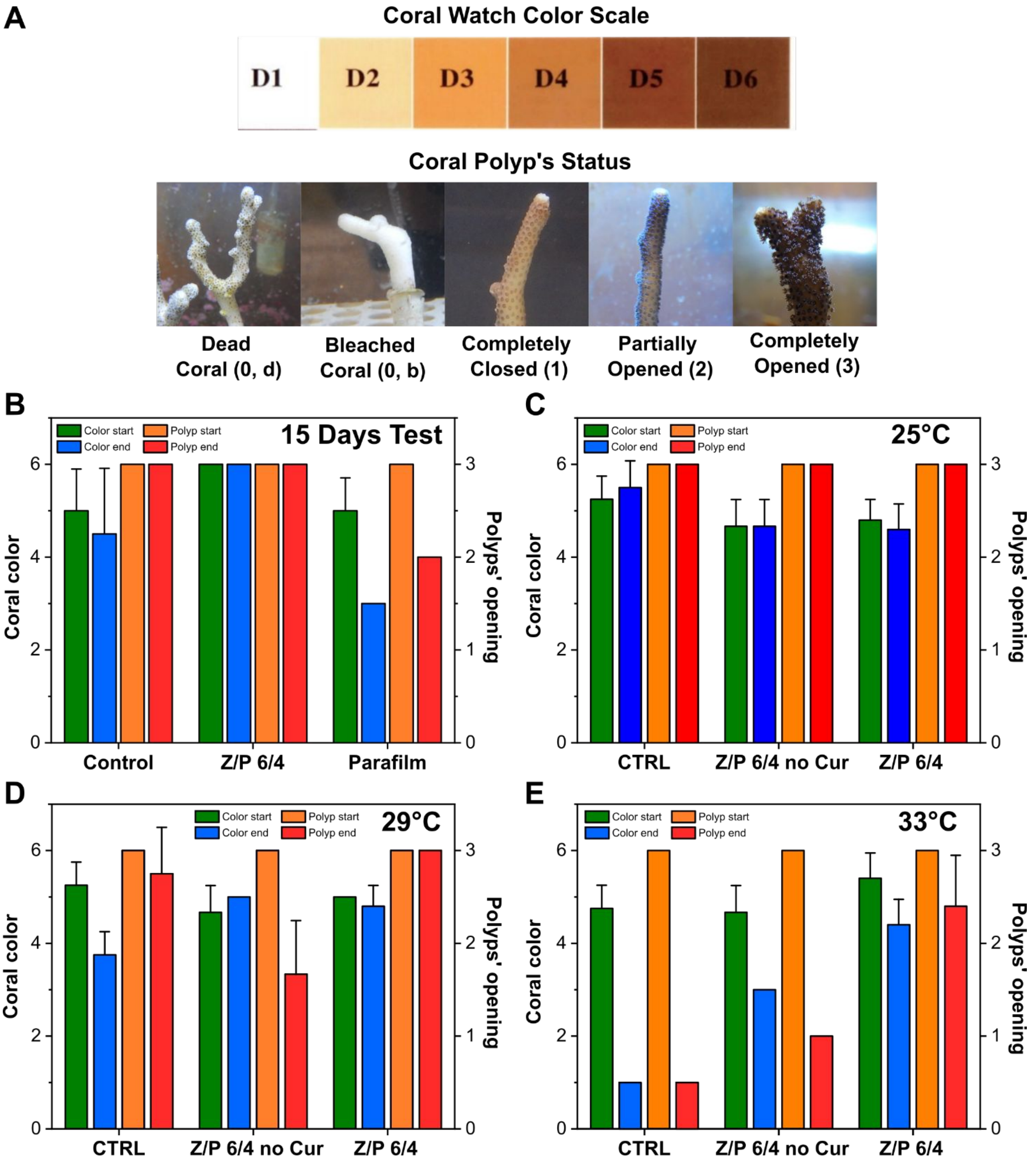

Figure S5. Color and polyps' opening. A, Coral color chart and polyps' opening status reference and codification. **B,** Color and polyps' opening for control coral, treated with Z/P 6/4 and Parafilm® after 15 days. **C, D, E,** Color and polyps' opening for control coral, treated with Z/P 6/4 no curcumin, and treated with Z/P 6/4 after 36 hours at 25, 29, and 33°C.

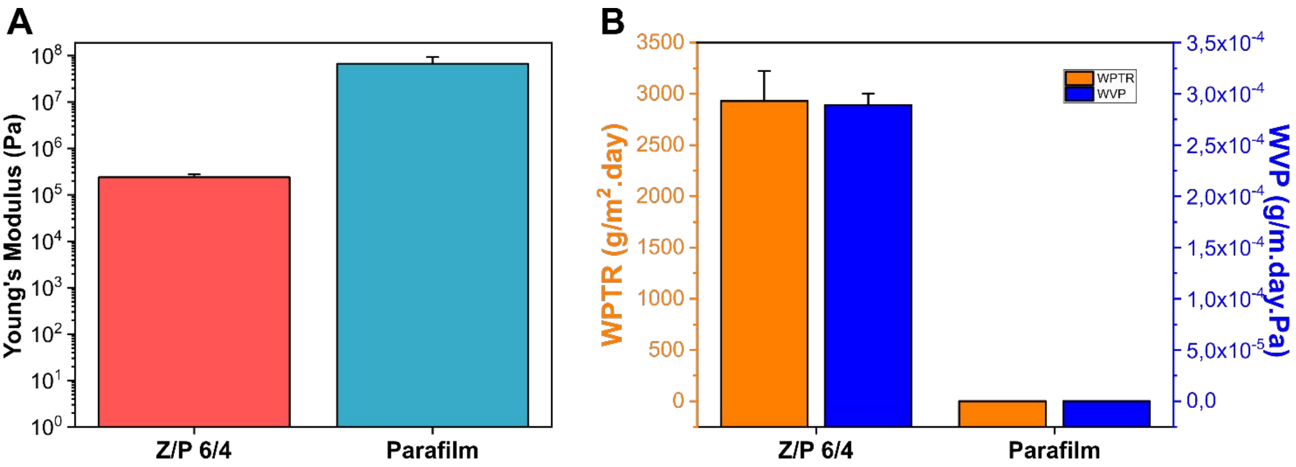

Figure S6. Comparison Z/P 6/4 and Parafilm materials. A, Results of the nanoindentation test for the Z/P 6/4 and Parafilm samples after immersion for 24 hours in seawater. **B,** Water vapor transmission rate and water vapour permeability values for the Z/P 6/4 sample and the Parafilm.

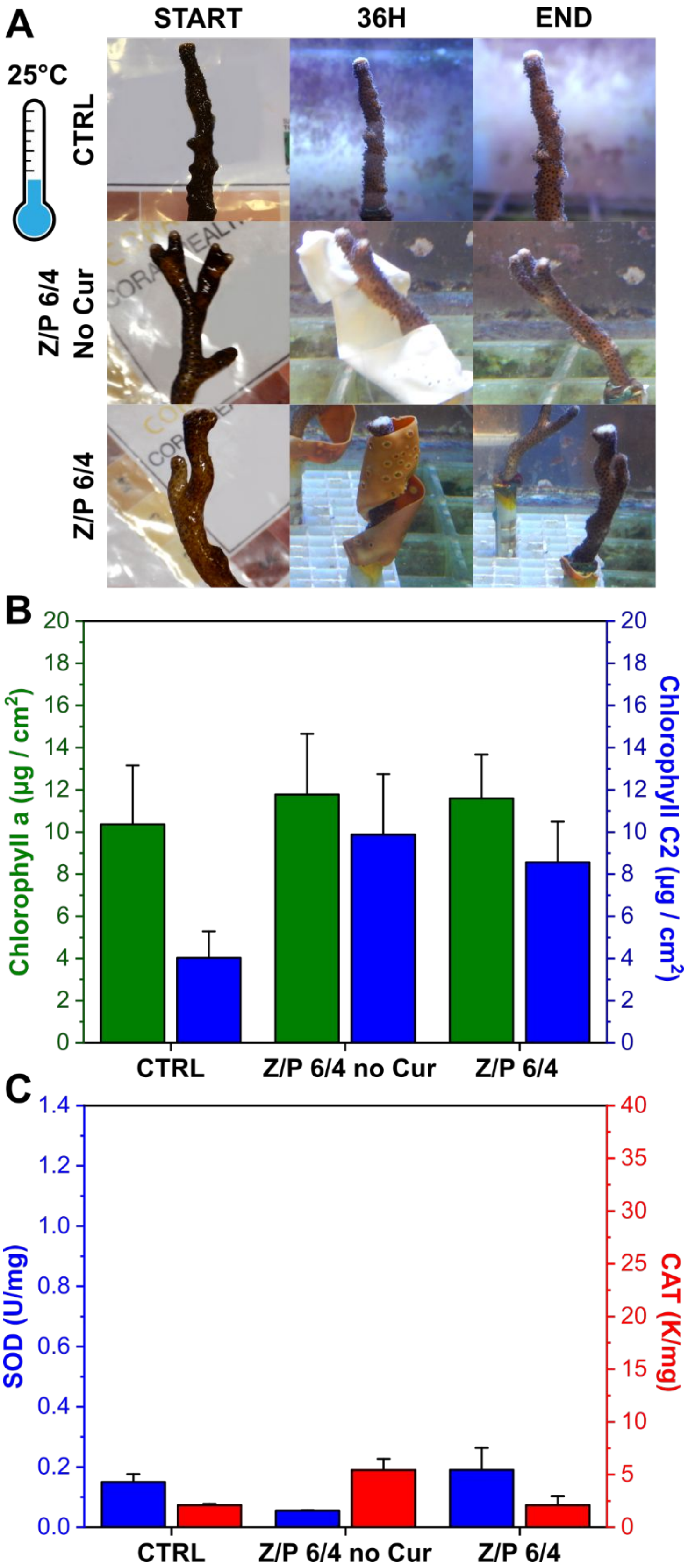
A
START
36H
END
25°C
CTRL
Z/P 6/4 No Cur
Z/P 6/4
B
Chlorophyll a (µg / cm2)
Chlorophyll C2 (µg / cm2)
0
2
4
6
8
10
12
14
16
18
20
CTRL
Z/P 6/4 no Cur
Z/P 6/4
C
SOD (U/mg)
CAT (K/mg)
0.0
0.2
0.4
0.6
0.8
1.0
1.2
1.4
0
5
10
15
20
25
30
35
40
CTRL
Z/P 6/4 no Cur
Z/P 6/4

Figure S7. Test at 25 °C. **A,** Photographs of untreated nubbin, treated with Z/P 6/4 without curcumin, and Z/P 6/4 at 25°C for 36 hours. **B,** Levels of chlorophyll *a* and *c2* for the control, Z/P 6/4 no curcumin, Z/P 6/4 samples at 25 °C. **C,** Levels of SOD and CAT for the control, Z/P 6/4 no curcumin, Z/P 6/4 samples at 25 °C.

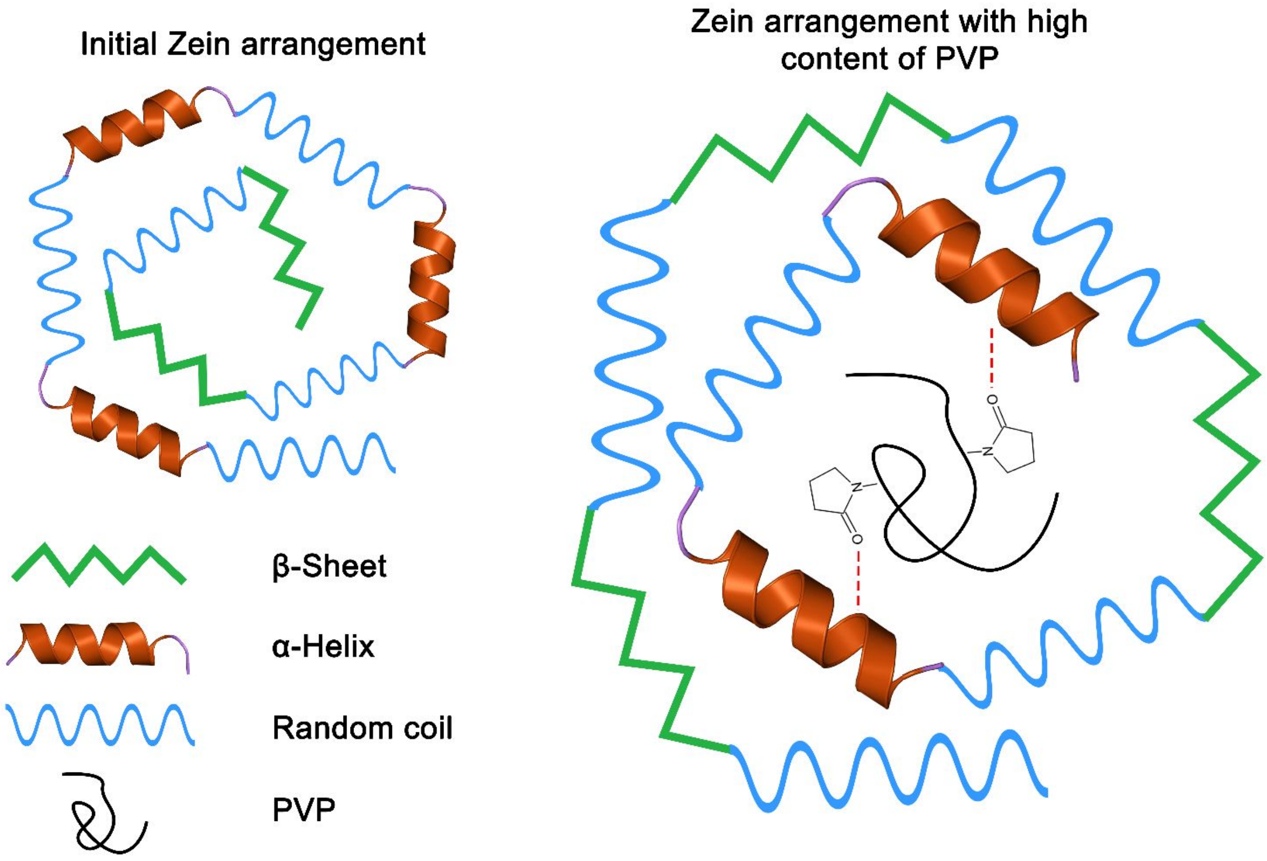

Figure S8. Zein/PVP assembly. Schematic representation of the supramolecular rearrangement of Zein. Initially, the α-helix hydrophilic domains are externally exposed. In the presence of high PVP content, the α-helix domains are moved internally, interacting with the synthetic polymer, while the β-sheets hydrophobic domains are externally exposed.

Table S1. Composition of the produced samples. Label and composition of the fabricated samples in terms of Zein and PVP % (w/w), Glycerol, and Curcumin % (w/w) with respect to total polymer weight.

Samples	Zein (w/w%)	PVP (w/w%)	Glycerol (w/w%)	Curcumin (w/w%)
Zein	100%	-	-	-
Zein + Gly	100%	-	10%	-
Z/P 10/0	100%	-	10%	2.4%
Z/P 9/1	90%	10%	10%	2.4%
Z/P 8/2	80%	20%	10%	2.4%
Z/P 7/3	70%	30%	10%	2.4%
Z/P 6/4	60%	40%	10%	2.4%
Z/P 5/5	50%	50%	10%	2.4%
Z/P 4/6	40%	60%	10%	2.4%
Z/P 3/7	30%	70%	10%	2.4%
Z/P 2/8	20%	80%	10%	2.4%
Z/P 1/9	10%	90%	10%	2.4%
Z/P 0/10	-	100%	10%	2.4%
PVP + Gly	-	100%	10%	-
PVP	-	100%	-	-

Table S2. Mechanical properties and water contact angle. The mean and the standard deviation of Young's Modulus, tensile stress at maximum load, elongation at break at R.H. 0 and 84%, and water contact angle values for all the zein and PVP-based samples.

	R.H. 0%			R.H. 84%			R.H. 44%
Sample	Young's Modulus (MPa)	Tensile Stress at Maximum Load (MPa)	Elongation at Break (%)	Young's Moduli (MPa)	Tensile Stress at Maximum Load (MPa)	Elongation at Break (%)	WCA (°)
Zein	789.6 ± 144.1	8.5 ± 3.8	1.4 ± 0.5	147.2 ± 41.5	3.8 ± 1.0	6.0 ± 1.6	54.2 ± 7.1
Zein+Gly	561.2 ± 63.9	11.3 ± 1.3	2.8 ± 0.4	94.6 ± 9.9	2.6 ± 0.5	7.6 ± 1.3	56.7 ±1.7
Z/P 10/0	644.1 ± 295.2	8.4 ± 5.0	1.8 ± 0.6	90.0 ± 22.9	2.9 ± 0.3	22.6 ± 8.1	45.2 ± 4.0
Z/P 9/1	515.9 ± 156.7	11.1 ± 2.5	3.9 ± 2.2	156.2 ± 34.7	4.8 ± 0.8	10.2 ± 2.2	43.4 ± 4.0

Z/P 8/2	608.3 ± 120.1	12.5 ± 2.5	2.9 ± 0.6	193.8 ± 50.1	6.3 ± 1.2	6.4 ± 0.5	51.1 ± 4.4
Z/P 7/3	717.2 ± 119.8	13.9 ± 2.0	2.9 ± 0.2	112.1 ± 42.4	3.7 ± 0.8	8.28 ± 0.8	59.8 ± 6.7
Z/P 6/4	356.0 ± 133.6	5.6 ± 2.5	4.2 ± 0.7	25.5 ± 8.8	1.1 ± 0.2	503.9 ± 85.6	104.3 ± 7.8
Z/P 5/5	350.1 ± 121.4	6.2 ± 2.3	4.7 ± 1.3	21.4 ± 0.6	1.3 ± 0.0	584.8 ± 54.0	112.6 ± 3.5
Z/P 4/6	380.1 ± 218.5	9.4 ± 4.1	5.5 ± 1.4	19.2 ± 5.8	1.4 ± 0.2	562.0 ± 45.9	106.5 ± 8.1
Z/P 3/7	447.8 ± 212.4	10.5 ± 5.2	5.2 ± 2.4	11.1 ± 2.4	1.3 ± 0.1	646.6 ± 42.7	109.0 ± 2.1
Z/P 2/8	404.5 ± 96.5	8.5 ± 2.5	3.1 ± 0.5	0.8 ± 0.1	1.0 ± 0.2	765.5 ± 45.1	108.8 ± 2.8
Z/P 1/9	634.8 ± 159.3	13.1 ± 3.1	3.2 ± 0.3	0.2 ± 0.1	0.9 ± 0.4	1037.7 ± 58.8	111.9 ± 3.5
Z/P 0/10	881.0 ± 143.2	16.8 ± 9.3	2.6 ± 1.3	0.3 ± 0.1	0.8 ± 0.2	935.7 ± 85.8	56.3 ± 6.9
PVP+Gly	507.0 ± 93.2	11.7 ± 2.7	3.6 ± 0.5	0.2 ± 0.0	0.5 ± 0.1	1423.8 ± 87.6	60.3 ± 3.9
PVP	1042.1 ± 223.0	29.3 ± 9.0	3.8 ± 0.7	0.3 ± 0.0	0.7 ± 0.1	1202.2 ± 61.5	61.0 ± 5.1
